# Untargeted Metabolomics Studies on Drug-Incubated *Phragmites australis* Profiles

**DOI:** 10.3390/metabo11010002

**Published:** 2020-12-22

**Authors:** Rofida Wahman, Andres Sauvêtre, Peter Schröder, Stefan Moser, Thomas Letzel

**Affiliations:** 1 Chair of Urban Water Systems Engineering, Technical University of Munich, Am Coulombwall 3, 85748 Garching, Germany; rofida.wahman@tum.de; 2 German Research Center for Environmental Health, Research Unit Comparative Microbiome Analysis, Helmholtz Centrum Munich, Ingolstadt Street 1, 85764 Neuherberg, Germany; andre.sauvetre@umontpellier.fr (A.S.); peter.schroeder@helmholtz-muenchen.de (P.S.); 3 Stefan Moser Process Optimization, Weberweg 3, D-83131 Nußdorf am Inn, Germany; stefan_moser@web.de; 4 Analytisches Forschungsinstitut für Non-Target Screening GmbH (AFIN-TS GmbH), Am Mittleren Moos 48, D-86167 Augsburg, Germany

**Keywords:** *P. australis* metabolic profile, untargeted metabolomics, diclofenac, carbamazepine, orthogonal partial least square-discriminant analysis (OPLS-DA)

## Abstract

Plants produce a huge number of functionally and chemically different natural products that play an important role in linking the plant with the adjacent environment. Plants can also absorb and transform external organic compounds (xenobiotics). Currently there are only a few studies concerning the effects of xenobiotics and their transformation products on plant metabolites using a mass spectrometric untargeted screening strategy. This study was designed to investigate the changes of the *Phragmites australis* metabolome following/after diclofenac or carbamazepine incubation, using a serial coupling of reversed-phase liquid chromatography (RPLC) and hydrophilic interaction liquid chromatography (HILIC) combined with accurate high-resolution time-of-flight mass spectrometer (TOF-MS). An untargeted screening strategy of metabolic fingerprints was developed to purposefully compare samples from differently treated *P. australis* plants, revealing that *P. australis* responded to each drug differently. When solvents with significantly different polarities were used, the metabolic profiles of *P. australis* were found to change significantly. For instance, the production of polyphenols (such as quercetin) in the plant increased after diclofenac incubation. Moreover, the pathway of unsaturated organic acids became more prominent, eventually as a reaction to protect the cells against reactive oxygen species (ROS). Hence, *P. australis* exhibited an adaptive mechanism to cope with each drug. Consequently, the untargeted screening approach is essential for understanding the complex response of plants to xenobiotics.

## 1. Introduction

Throughout human history, plants have been the most important source for pioneering medicines, flavors and industrial materials. Plants produce up to 200,000 natural products with a vast chemical diversity using a wide range of enzymes and substrates [1]. Among them, primary metabolites are relatively few and are defined as fundamental to plant physiology. In contrast, secondary metabolites (specialized metabolites) are essentially diverse. The high diversity of specialized metabolites leads to functional variation. Specialized metabolites play important roles in the interactions between the plant and its environment since they are involved in protection against environmental stress, competition or pollinator attraction and some are involved in vegetative or floral development. Furthermore, they play a vital role in the conception of environmental signals and their translation in biography traits. Therefore, they may play major roles in allowing the organism to sustain environmental constraints.

Among many other uses, plants have been used to restore environmental quality via adsorption and accumulation of organic xenobiotics (such as pesticides, dyes, drugs, etc.) inside their tissues. In the process of phytoremediation, plants can degrade these external compounds through a specific enzymatic scaffold consisting of three phases [2,3]. In this process, they utilize enzymes like cytochromes P450 or peroxidases to activate the xenobiotics in phase I and conjugating enzymes as glucosyl-transferases, malonyl-transferases or glutathione S-transferases in phase II for detoxification [4,5].

Diclofenac (DCF) and carbamazepine (CBZ) belong to the problematic xenobiotics in European wastewater treatment plants since they can harm the ecotoxicological equilibrium. They have not only been detected in wastewater but also in biosolids and wastewater effluents [6,7,8]. Recently, the maximal measured concentrations of DCF and CBZ in municipal wastewater has ranged between 440 and 7100 ng · L^−1^ and 1075–6300 ng · L^−1^ of, respectively [9,10]. DCF is one of the most commonly used non-steroidal anti-inflammatory drugs, while CBZ has been used widely as an antiepileptic and mood stabilizer since the 1970s. Both DCF and CBZ are taken up and translocated into the aerial parts of plants, where they can be accumulated or metabolized into more or less toxic products [7,11].

One of these species is *P. australis*, known as “common reed”. *P. australis* belongs to the Poaceae family and is an invasive plant that spreads worldwide [12]. It has been used for a long time in wetlands to remove pollutants, reduce nitrogen loads and provide oxygen to the rhizosphere [5,13]. Furthermore, it is used as a resource for traditional crafts and fodder. In some regions like Northern China, it is grown as a crop and its leaves are used in the treatment of bronchitis and cholera [12].

The chemical diversity (such as polarity, stability, reactivity or ionization) of plant molecules prohibits investigation of the metabolites´ full picture and understanding changes in their occurrence. The analysis purpose and chemical nature of plant metabolites determine the analytical technique. Also, the untargeted concept demands an analytical device, which can separate different classes of metabolites with a wide range of polarities. For untargeted metabolite analysis, several analytical techniques are available. These approaches apply liquid chromatography-mass spectrometry (LC-MS) [14], gas chromatography-mass spectrometry (GC-MS) [15] or nuclear magnetic resonance (NMR) [16] to analyze a large number of different chemical metabolites classes within one single analysis. NMR has analytical reproducibility and a non-destructive nature; however, it has relatively low sensitivity compared to MS. Besides, a mass spectrometric untargeted screening strategy can identify a large number of molecules independently to provide a preferably holistic picture of the plant’s metabolome. Also, the thermal stability of the stationary phase, metabolites and their derivatives, which might introduce artifacts, limit the metabolome coverage derived by GC-MS. Thus, the usage of LC-MS has expanded rapidly over the past ten years in untargeted metabolomics analysis [17].

The serial coupling of reversed-phase liquid chromatography (RPLC) and hydrophilic interaction liquid chromatography (HILIC) is often used to separate compounds with differing polarities in a single run [18]. Further, connecting the serial RPLC-HILIC coupling with an accurate high-resolution time-of-flight mass spectrometer (HRMS) provides the detection of a wide range of metabolites.

Recently, the awareness of untargeted metabolomics analysis has increased due to its capabilities in the assessment of xenobiotics exposure/specific biomarkers and the risk of contaminants to living organisms. For example, the metabolic fingerprints of *Plantago lanceolata* showed various chemical changes as a response to different stresses [19].

Metabolic fingerprinting experiments aim to determine relative differences between two or more systems elucidating a biological relationship. Therefore, statistical strategies are typically used in a chemometrics style. Univariate and multivariate statistics can be used as standard approaches to extract relevant information from complex datasets [20,21,22,23].

This study aims to investigate the effect of DCF, CBZ and their transformation products on *P. australis* metabolites using mass spectrometric untargeted screening analysis. By studying the fingerprint of *P. australis* leaves, rhizomes and roots with/without incubation at two different concentrations, 10 and 100 µM and 10 and 50 µM of DCF, as well as CBZ, respectively. Also, a statistical workflow was used to discriminate against the changes in *P. australis* metabolites and detect the differentiating metabolic profiles.

## 2. Results

Diclofenac and carbamazepine are considered problematic environmental pollutants because they can be found in surface waters (mainly in wastewater treatment plant effluents) in high concentrations. Several plant species have been found capable of absorbing and detoxifying them. Despite this, the effects of diclofenac and carbamazepine and their transformation products on plant metabolic profiles need more investigation. Consequently, *P. australis* was incubated with 10 and 100 µM DCF and with 10 and 50 µM CBZ, respectively. Each plant (i.e., leaves, rhizomes and roots, respectively) was extracted with four different solvents. The different samples were injected into a robust and reproducible serial coupling RPLC-HILIC in hyphenation with electron spray ionization- time of flight- mass spectrometer (ESI-TOF-MS). Both robustness and reproducibility of the analytical system was checked and proven with a mixture of 13 reference standards (Tables S1 and S2). The statistical evaluation discriminated between the different extraction solvents, plant parts and incubation. Moreover, the statistical analysis was enabled to assign the changes in *P. australis* metabolic profiles and the differentiating metabolic profiles (DMF) between the control and incubated samples. Furthermore, different DCF and CBZ transformation products could be identified or suspected. Thus, changes in *P. australis* biosynthetic pathways could be predicted.

### 2.1. Metabolic Profiling Elucidation in Phragmites australis Extracts with RPLC-HILIC-ESI-TOF-MS

A mixture of 13 reference standards was injected at the beginning/end and fixed intervals during the experimental sets (i.e., after each extract batch). The results of the standard mixture proved the accuracy, repeatability and reproducibility of the analytical system. Mass and RT of the standards during the experiment showed an acceptable deviation of less than 8 ppm (with a ToF system from the year 2012) and % RSD less than 2 except for gabapentin, carbetamid and sotalol. For more information, readers are referred to Wahman et al., 2019 [18].

**Figure 1:**
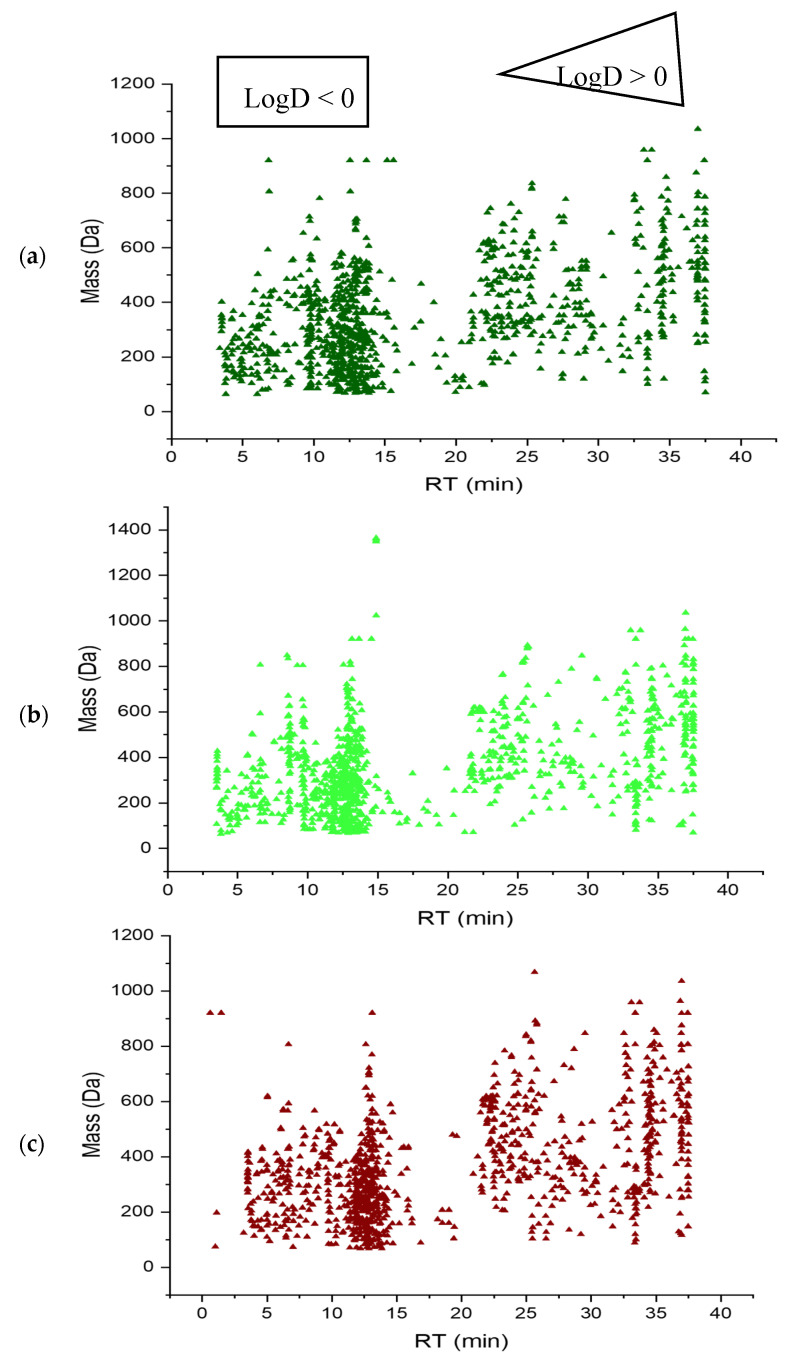
Retention time (RT)/Mass plot of *Phragmites australis* 100% Methanol extracts analyzed by RPLC-HILIC-ESI-TOF-MS in positive electrospray ionization mode. (**a**) Leaf; (**b**) Rhizome; (**c**) Root, which showed the features’ separation according to their polarity and detected according to their *m/z*.

The four different extracts 100% MeOH, acidic 90% MeOH, 50% MeOH and 100% H_2_O of *P. australis* leaf, rhizome and root were analyzed similarly with RPLC-HILIC-ESI-TOF-MS coupling as previously described [24,25,26]. The obtained mass spectrometric total ion chromatograms (TICs) were interpreted to extract the feature (extracted masses, RT and signal abundance) according to the parameters mentioned in Section 4.6.1. Background signals (i.e., all the peaks detected in the corresponding blank) were deleted to avoid false positives. The Retention Time (RT)/Mass plots of the different background were presented in (Figure S1). Lastly, the features found in the triplicate injections were considered for further analysis. Exemplarily, Figure 1 represents (RT)/Mass plots for 100% MeOH extracts of *P. australis* leaf, rhizome and root in positive ion mode. The highly polar to polar compounds eluted at RT < 15 min, with logD values below zero (HILIC part). The nonpolar compounds were eluted at RT > 15 min, with logD values above zero (RPLC part). The differences in chromatographic fingerprints reflected the variability in metabolite profiles (and composition) in the leaf, rhizome and root samples. Detailed information and a description of data analysis have already been discussed in previous publications [18,26].

### 2.2. Different Extracts of Phragmites australis’s Metabolic Fingerprints

**Figure 2:**
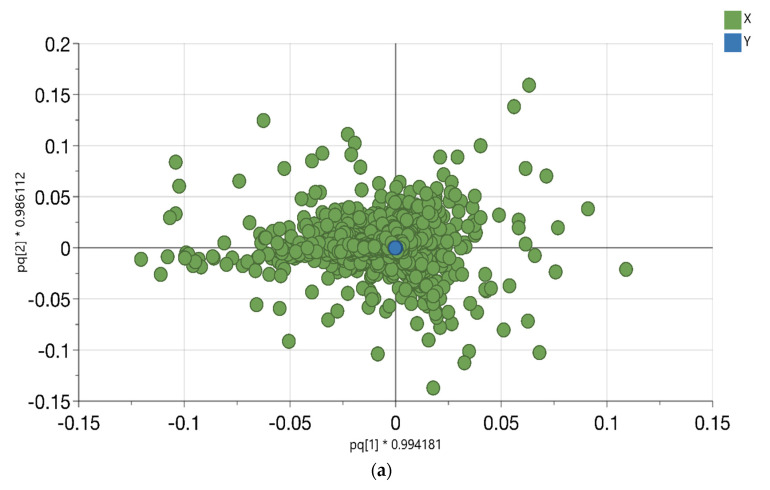

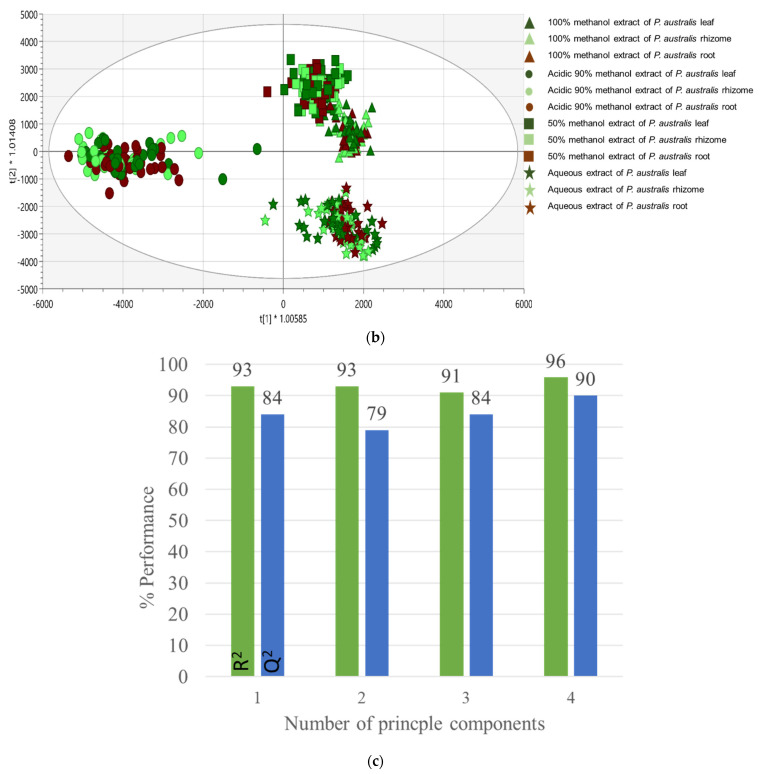

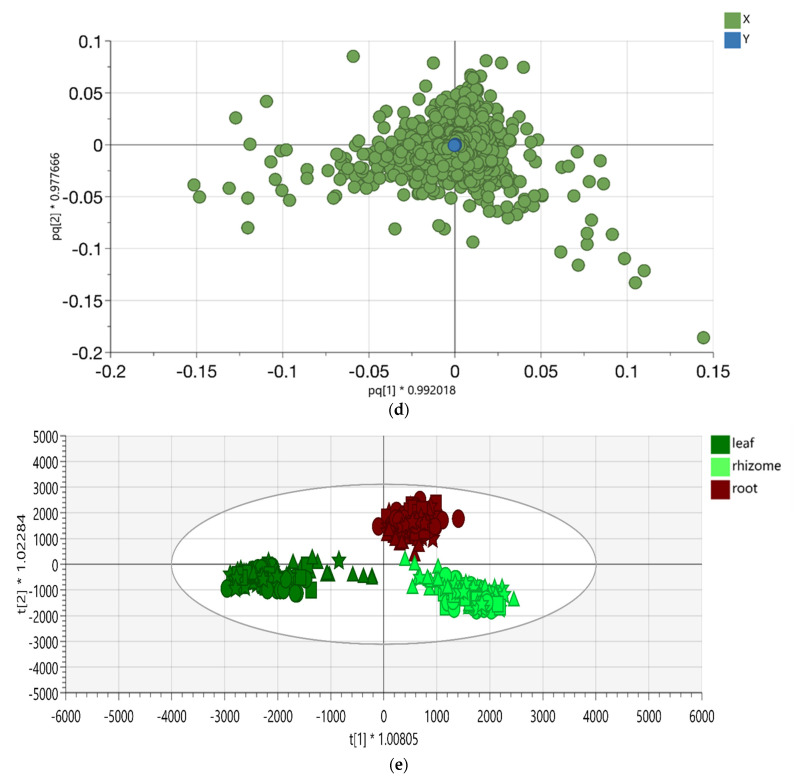
(**a**) The OPLS-DA score plot of different *Phragmites australis* extracts with a confidence limit of 95% discriminating according to the solvent used in the extraction. The variables were plotted according to the first principal component (t1) and the orthogonal component (t2). The triangles represent 100% methanol extracts, circles represent acidic 90% methanol extracts, the squares represent 50% methanol extracts and the stars represent aqueous extracts, respectively. The green color represents leaf samples, the light green color represents rhizome samples and the brown color represents root samples, respectively. Each symbol represents one observation of *P. australis* leaf, rhizome and root plant part; (**b**) loading scatter plot for the selected principal components; (**c**) the Q^2^/R^2^ Overview plot displays the individual cumulative R^2^ (green columns) and Q^2^ (blue columns) for the goodness of fits and cross-validation parameters; (**d**) the loading scatter plot for the selected principal components displays the relation between the different *Phragmites australis* samples and the chosen metabolites; (**e**) the OPLS-DA score plot of the different parts of *Phragmites australis* with a confidence limit of 95%, discriminating according to the plant part.

Untargeted metabolomics analysis demands minimal pretreatment methods to allow the detection of almost all the sample metabolites. Different leaf, rhizome and root samples were extracted with the described four solvents (i.e., 100% MeOH, acidic 90% MeOH, 50% MeOH and 100% H_2_O), individually, which ensured the extraction of a wide range of metabolites from nonpolar to highly polar metabolites relative to the solvents, which were used in the extraction process. All the extracts were analyzed along a metabolites’ fingerprint strategy. In orthogonal partial least square-discriminant analysis (OPLS-DA), the variables were *P. australis* metabolites, which were plotted in the loading score plot (as in Figure 2a). The OPLS-DA model described the variables according to the solvents of the extraction class. Of the data variations, 18.3% (R^2^X(cum)) are responsible to distinguish between the classes that were previously established based on solvents. The rest of the variation (orthogonal components) describes the variation within the solvent classes. The high value of those parameters indicates that the OPLS-DA model had a good classification and prediction efficiency to distinguish between different extracts, even though it described one variation (i.e., solvent type). This is owing to the accuracy of variables, which were separated with a robust and reproducible LC-system. In (Figure 2b), the samples are distributed according to t1 (predicative component) and t2 (orthogonal component). The predictive component (t1) separated the samples into two groups, the first group (negative side) contained the acidic 90% MeOH extracts and the second group (positive side) consisted of 100% MeOH, 50% MeOH and 100% H_2_O extracts (Figure 2b). Moreover, the orthogonal component (t2) described the differences within the group. Consequently, it separated the second group into 100%MeOH and 50%MeOH in the positive part and the 100% H_2_O extracts in the negative part (Figure 2b). Further, in (Figure 2c), good separation was reached according to the cumulative goodness of fit and the cross-validation parameters of each variable R^2^ and Q^2^, respectively. Therefore, the model had no risk of overfitting.

### 2.3. Phragmites australis Leaf, Rhizome and Root Metabolic Fingerprints

In this section, the 11,442 variables were differentiated according to the plant part that they originated from. The variables were in the loading score plot (Figure 2d). This step was performed to improve the statistical significance of the dataset through additional cross-validation. Also, the OPLS-DA analysis was conducted to investigate the structure of the data. Another 18% of the data was investigated through the variation between X (metabolites) and Y (plant parts) given by R^2^X(cum) and explained approximately 94% of the variations in the various samples (R^2^Y(cum)). We found the predictive value of the model was (Q^2^(cum) = 74%), which was created by OPLS-DA. The cross-validation performance was confirmed by analysis of variance (ANOVA). OPLS-DA discriminates the different samples of *P. australis* leaf, rhizome and root regardless of incubation, with or without DCF or CBZ and regardless of the extraction solvent composition according to the plant part (Figure 2e).

The predictive component t1 differentiated between leaf extracts in one group (negative part) and the root and rhizomes extracts in the second group (positive part), as shown in (Figure 2e). However, the t2 (orthogonal component) differentiated rhizomes sample in the negative part and the roots in the positive one.

The quality of the OPLS-DA module was expressed by the cumulative value of the goodness of fit and the cross-validation for each value R^2^ and Q^2^, respectively, as shown in the Q^2^/R^2^ Overview plot (Figure S2A). The high values of the previous parameters indicated a good classification and prediction efficiency to distinguish between different plant parts.

### 2.4. Untargeted Metabolomics Analysis of Phragmites australis Incubated with DCF or CBZ

**Figure 3:**
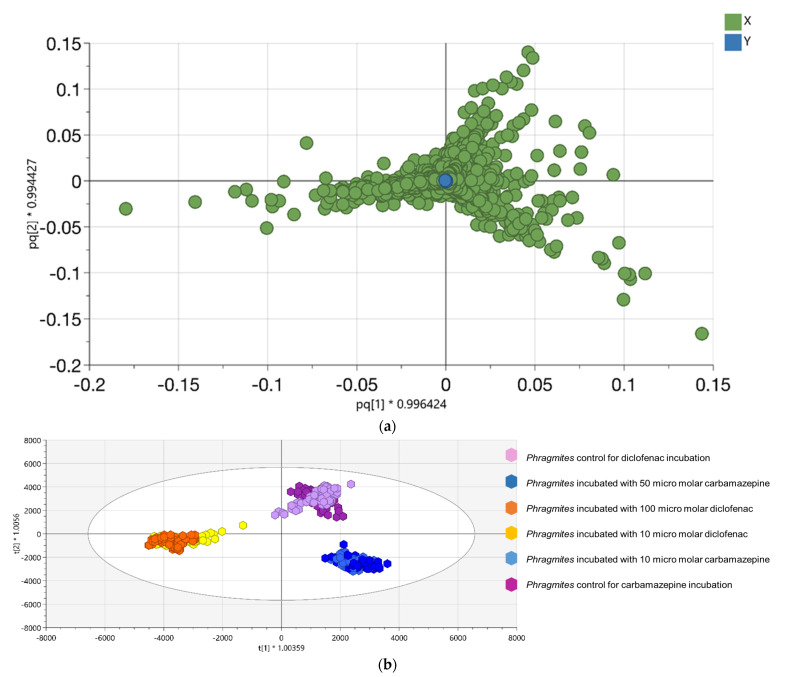
(**a**) The loading plot displays the relation between the different *Phragmites australis* samples and the chosen metabolite; (**b**) the OPLS-DA score plot of different *Phragmites australis* samples incubated with 10 and 50 μM carbamazepine, 10 and 100 μM diclofenac, individually. The confidence limit is 95%. For carbamazepine incubation the purple color represents the control group, the light blue represents a sample incubated with 10 μM carbamazepine and the blue color represents samples incubated with 50 μM carbamazepine. For diclofenac incubation the light purple color represents the control group, the yellow color represents samples incubated with 10 μM diclofenac and the orange color represents samples incubated with 100 μM diclofenac.

**Figure 4:**
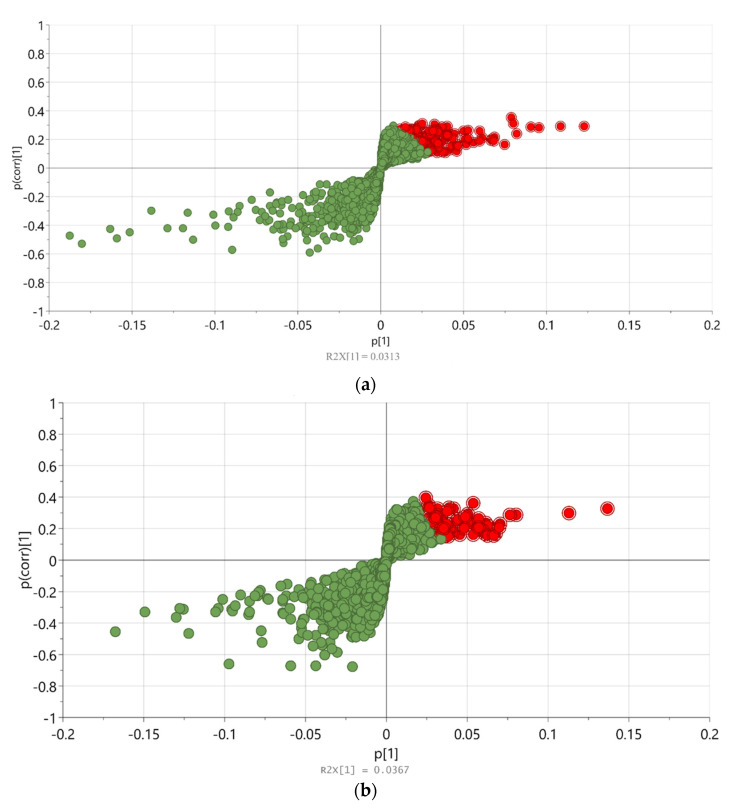

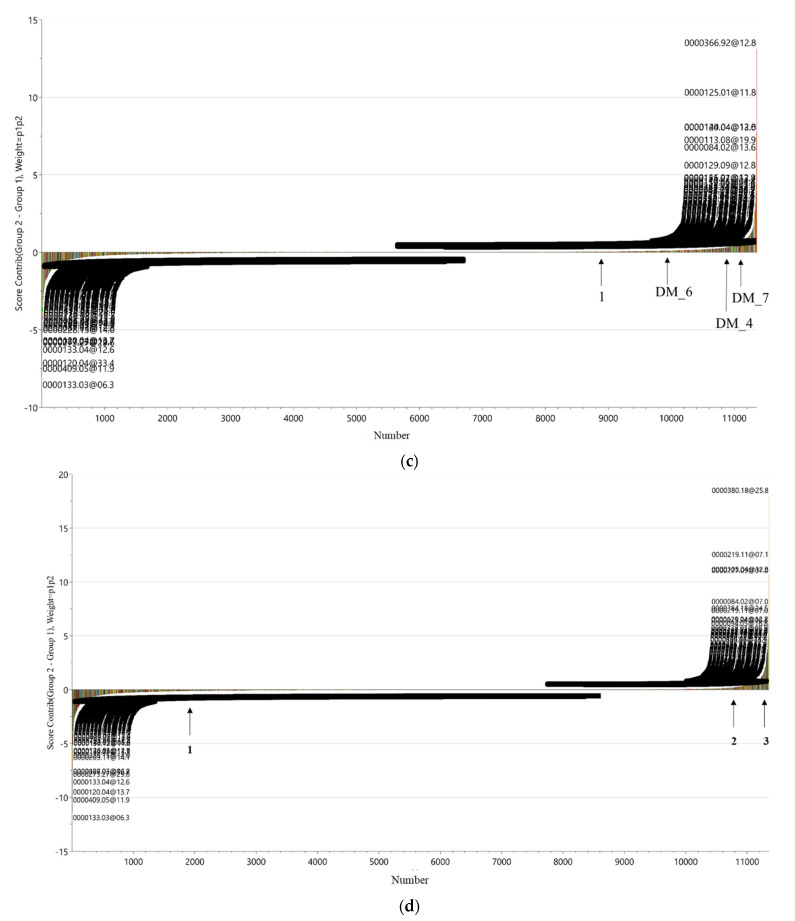
(**a**) S-plot of *Phragmites australis* control and incubated with 10 and 100 μM diclofenac samples; (**b**) S-plot of *Phragmites australis* control and incubated with 10 and 50 μM carbamazepine samples. The S-plot provides the visualization of the loading components (green color) of OPLS-DA to enable the interpretation of the data. The red-labeled compounds represent the differentiating metabolic profile (DMF) of each incubation; (**c**) the contribution plot shows the up- and down-regulated compounds due to the incubation of *Phragmites australis* with 10 and 100 μM diclofenac, individually. Down-regulated compounds have negative values, while up-regulated compounds have positive values. (1) Quercetin, DM_6, DM_4 and DM_7 have up-regulated in *Phragmites australis* due to incubation with diclofenac at (8820, 0.0385581), (9968, 0101411), (10,817, 0.279365) and (10,985, 0.389), respectively; (**d**) the contribution plot of *Phragmites australis* with 10 and 50 μM carbamazepine, individually. Down-regulated compounds have negative values as compound 1 which was quercetin at (2033, −0.118706). Up-regulated compounds have positive values as compounds 2 and 3 which were 2,3-dihydro-2,3-dihydroxycarbamazepine at (10,921, 0.288274) and carbamazepine-10,11-epoxide at (11,270, 1.27941), respectively.

The untargeted metabolomics analysis of *P. australis* incubated with/without DCF or CBZ, respectively, was performed using OPLS-DA to assign the changes in its metabolic fingerprint. The *P. australis* metabolic fingerprints of different extraction solvents with various plant parts were investigated in Section 2.2 and Section 2.3, respectively, to test the organization and reliability of the data. Then, the large data set was used to perform the untargeted analysis and assign a list of metabolites that determined the distance between different groups. Also, the metabolic markers of *P. australis* incubated with/without DCF or CBZ were plotted each by the OPLS-DA, which represented the variability in metabolic patterns due to the different incubation (Figure 3, that is, the loading plot in Figure 3a and the OPLS-DA score plot in 3B). OPLS-DA analysis showed the identified and unidentified metabolites, which distinguished the different clusters according to the characteristic change of control or incubated samples metabolite profiles. It was used to enhance the quality of pairwise classification analysis. The relative high goodness of fit indicated the good separation of different incubation groups of *P. australis* R^2^ and Q^2^, respectively, (Figure S2B). To facilitate the interpretation and visualization of OPLS-DA, the S-plot was drawn to illustrate the model’s influence with accuracy in the search for differentiating metabolic profile (DMF). S-plot analysis represented the highest contributing signals for the control and incubation of *P. australis* with DCF or CBZ (Figure 4a,b, respectively). The DMF was extracted from S-plots, which were marked in red color. They were selected based on their contribution to the variation and correlation within the data set between the X-variables and the predictive component t1 (p (corr) vector). Hence, they were considered functionally in the combined form of a metabolic profile, which was distinguished between the control and the incubated *P. australis*. In Figure 4c,d, the contribution plots extend the data to better visualization and indicate regions responsible for sample clustering [22]. The contribution plot summarized the changing trends in the metabolites in the pairwise groups (i.e., control and incubated samples) through expressing the fold change of the metabolites between the control and incubated samples. As examples, succinic acid (DM_4), propane-1,2,3-triol (DM_6) and 2- hydroxypropanoic acid (DM_7) were detected as transformation products of DCF (DMs, see Table 1), thus they appeared significant in the positive part of the plot, (Figure 4c). Also, quercetin (compound 1 in Figure 4c,d) was found in elevated signal heights after *P. australis* exposure to DCF. Quercetin is a flavonoid that protects the plant against reactive oxygen species (ROS) during exposure to pharmaceuticals.

In CBZ incubation, the contribution plot showed that in incubated samples 2,3-dihydro-2,3-dihydroxy-carbamazepine (compound **2** in Figure 4d) and carbamazepine-10,11-epoxide (compound **3** in Figure 4d) could be identified as metabolites (evaluated and proven with reference standards) and appeared with significant intensity. The carbamazepine-10,11-epoxide was statistically significant variable important (VIP) > 1 and *p* < 0.05. However, in this case quercetin (compound **1** in Figure 4d) intensity was found to decrease significantly.

### 2.5. Metabolism of Diclofenac in Phragmites australis

*P. australis* was exposed for 96 h to diclofenac in the two concentrations of 10 and 100 µM, respectively. Thereafter, the data was processed with MassHunter Workstation Software Profinder B.06.00 (Agilent Technologies, Waldbronn, Germany) to detect the DCF molecule and its metabolites (including background subtraction). Diclofenac and its hydroxylated metabolites were detected in the roots and rhizomes of *P. australis*. Diclofenac was identified at 295.0173 Da with 2 ppm deviation from the monoisotopic mass, which eluted at 26 min (logD (pH7) > 0). The EICs of diclofenac in extracts and standard are shown in Figure S3. Further, hydroxylated metabolites were suspected according to the previously mentioned criteria at 311.0114 Da with 0.6 ppm deviation from the monoisotopic mass, which eluted at 24 min (logD (pH7) > 0) [27]. Moreover, the analysis of different extracts of *P australis* samples based on the mass, RT and LogD (pH7) revealed seven proposed metabolites of diclofenac, as summarized in Table 1 and Figure S3. The comparison of mass spectra from leaf, rhizome and root resulted in evidence for five metabolites of diclofenac in all parts of the treated samples of *P. australis*. However, DM_6 and DM_7 were detected in leaf extracts (Table 1).

### 2.6. Metabolism of Carbamazepine in Phragmites australis

*P. australis* was exposed for 96 h to carbamazepine in two CBZ concentrations of 10 and 50 µM, respectively. Thereafter, the data was processed with MassHunter Workstation Software Profinder B.06.00 to detect the compound and its metabolites (including background subtraction). According to Sauvêtre and co-workers (2018), four different pathways for carbamazepine metabolism (PCM) were investigated in the plant [28].

The different transformation products were identified in different *P. australis* samples using reference standards. The mean monoisotopic mass of standards and different samples and their absolute variation were tabulated, as well as the retention time in both standards and samples and the variation between them (Table S3, Figure S4). Carbamazepine-10,11-epoxide, 10,11-dihydro-10,11-dihydroxy-carbamazepine, 10,11-dihydro-10-hydroxy-carbamazepine, 9-acridine carboxaldehyde and 2,3-dihydro-2,3-dihydroxy-carbamazepine were identified in *P. australis* incubated samples. The mass deviation was less than 5 ppm and the deviation in the RT was less than 0.3 min. Carbamazepine-10,11-epoxide and 9-acridine carboxaldehyde have been identified in leaf, rhizome and root in both incubation levels 10 and 50 µM. 10,11-dihydro-10,11-dihydroxy-carbamazepine and 2,3-dihydro-2,3-dihydroxy-carbamazepine were identified in root and rhizome extracts in both incubation concentrations. Moreover, the first was identified in leaf extracts of 50 µM carbamazepine; however, the latter was not detectable in leaf extracts, respectively.

### 2.7. Impacts of DCF and CBZ on Phragmites australis Metabolic Pathways

Metabolic pathway analysis was performed to identify the pathways that were induced upon the incubation of *P. australis* with DCF or CBZ via MetaboAnalyst 4.0 software (Montreal, QC, Canada) based on the Kyoto Encyclopedia of Genes and Genomes (KEGG) database. The results are established on OPLS-DA and S-plot analysis of different metabolites with/without DCF or CBZ incubation depending on the p (corr) vector in the OPLS-DA module. Consequently, these metabolites represented the metabolic profile (DMF) which differentiate between control and incubated samples.

For the investigation, the mummichog algorithm was the first implementation of this concept to infer pathway activities from a ranked list of MS peaks identified by untargeted metabolomics. The DMF was applied to test their participation in pathways using a combination of network analysis and functional enrichment analysis and listed in Tables S4 and S5. The pathways exhibiting *p* < 0.05 were considered a statistically significant metabolic pathway, meaning that they were affected with DCF or CBZ or their transformation products via MetaboAnalyst 4.0 software based on the Kyoto Encyclopedia of Genes and Genomes (KEGG) database.

**Figure 5:**
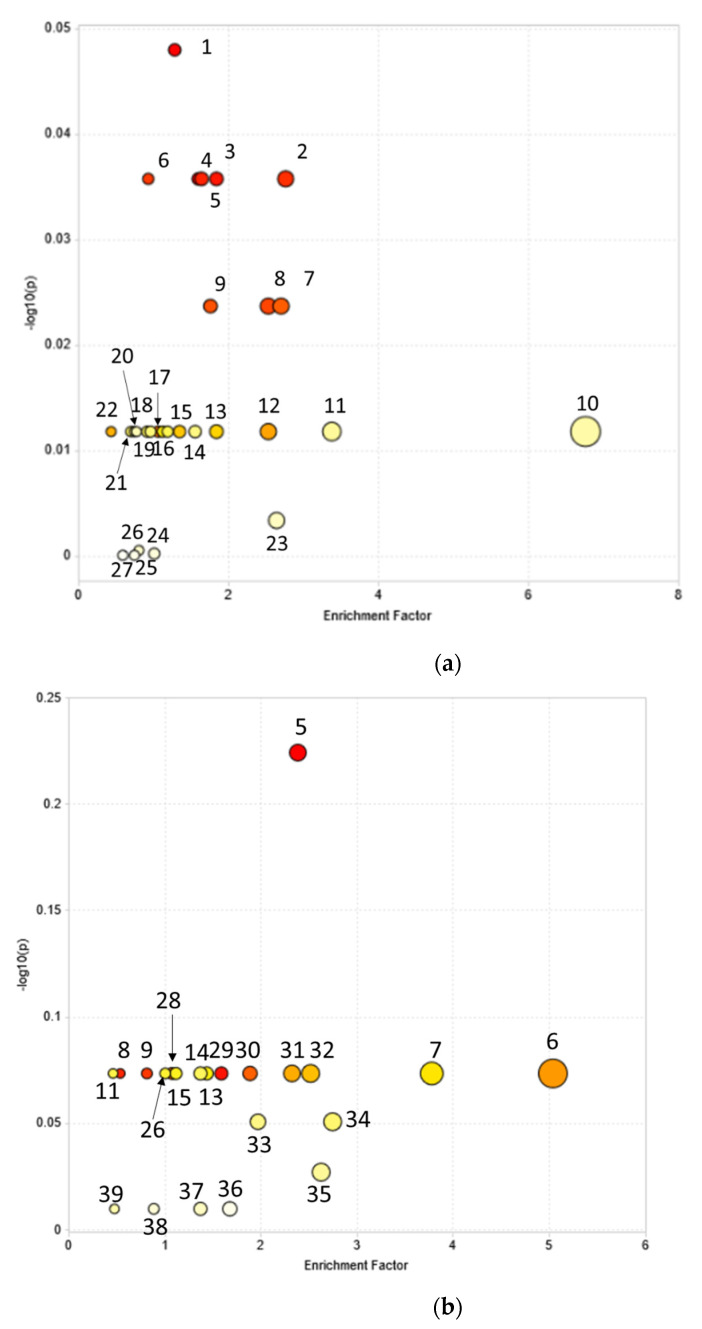

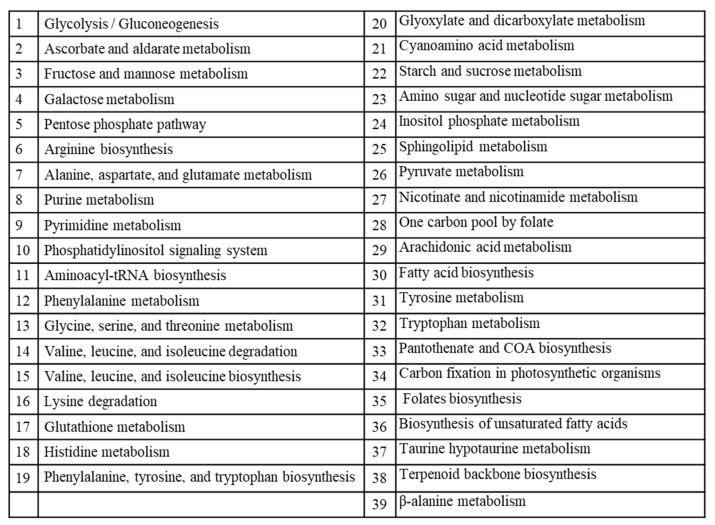
Overview of pathway analysis using MetaboAnalyst 4.0. For metabolite set enrichment analysis of *Phragmites australis* differentiating metabolic profile (DMF) after incubation with (**a**) 10 and 100 μM diclofenac; (**b**) 10 and 50 μM carbamazepine. The overview displays all matched pathways as circles. The color and size of each circle are based on the *p* value and the impact of the pathway value, respectively. The names of the metabolic pathways are listed in the table.

KEGG pathway analysis of the analyzed metabolites using the pathway data set of *A. thaliana* matched 27 pathways in *P. australis* incubated with DCF. Further, 11 were significantly biologically active and are listed in the table in (Figure 5a). They were identified as glycolysis/gluconeogenesis, ascorbate and aldarate metabolism, fructose and mannose metabolism, galactose metabolism, the pentose phosphate pathway, arginine biosynthesis, alanine, aspartate and glutamate metabolism, purine metabolism, pyrimidine metabolism, glutathione metabolism and phenylalanine metabolism.

After CBZ incubation, 22 pathways were significantly altered. Twelve pathways have a more significant *p* value, relating them to the effect of CBZ and/or its transformation products (Figure 5b). They were the pentose phosphate pathway, purine metabolism, pyrimidine metabolism, fatty acid biosynthesis, arachidonic acid metabolism, tyrosine metabolism, tryptophan metabolism, β-Alanine metabolism, arginine and proline metabolism, pantothenate and CoA biosynthesis, carbon fixation in photosynthetic organisms and folate biosynthesis. The previous pathways were induced after incubation with DCF or CBZ.

## 3. Discussion

*P. australis* metabolic profiles were investigated in different samples with RPLC-HILIC-ESI-TOF-MS. The system accuracy and reproducibility were checked using a mixture of 13 reference standards. The results of the quality control mixture revealed that the serial coupling produces robust and reproducible data, which was used for further statistical analysis. *P.australis* metabolomics’ statistical analysis of the four different extracts reveals that the 90% acidic MeOH extracts are very different from the other extracts of various *P. australis* leaf, rhizome and root samples. However, the 100% MeOH, 50% MeOH and 100% H_2_O extracts are more related to each other. 100% MeOH was generally located in the middle between 50% MeOH and 100% H_2_O extracts, however, representing a similar solution behavior to all three extract types. A large t2 range value was observed mostly in acidic 90% MeOH extracts, that is, a value far above the critical limits in the score space. Hence, this is likely to be an outlying observation. The elimination of acidic 90% MeOH extracts might improve the model. Also, most of the metabolites extracted with acidic 90% MeOH have been detected in three other solvents. The same observation was made for *Lemna minor* extracts and can be described with the different occurrence of charged molecules caused by different pH values [26].

The statistical software allowed cross-validation between the datasets. Furthermore, an additional step of cross-validation of the data was done through the differentiation between the x (metabolites) and the plant parts. The results reveal the significance of the data organization. Hence, the compounds extracted from roots and rhizomes are more correlated than compounds in leaves, which supports the physiological similarities between them [29]. After the additional cross-validation step, the OPLS-DA model was built to assign the variables, which differentiate between control and incubated samples. OPLS-DA is a supervised approach that tends to improve the separation between (two or more) groups of samples. For this reason, it is widely used for classification purposes and biomarker identification and/or differentiating metabolic profiles (DMF) in metabolomics studies [21].

The DMF of *P. australis* was extracted. Further, the fold change of the different variables in the control and incubated samples show an increase in quercetin in DCF incubation; however, in this case of CBZ, it was found to decrease significantly. *P. australis* responds to the incubation with different classes of compounds, with the potential to protect it against ROS, such as polyphenols and flavonoids. Also, it might be that CBZ does not cause oxidative stress to the same extent as DCF. Furthermore, succinic acid (DM_4), propane-1,2,3-triol (DM_6) and 2-hydroxypropanoic acid (DM_7) appeared significant in the positive part of the plot with VIP > 1 and *p* < 0.05 (Figure 4c). They were shifted toward the middle of the figure more than CBZ transformation products, indicating that they originated partially from diclofenac. The same was in CBZ transformation products, which were not produced by *P. australis.* The carbamazepine-10,11-epoxide was statistically significant: VIP > 1 and *p* < 0.05. Carbamazepine-10,11-epoxide is considered a main transformed product of carbamazepine in plants (compound **3** in Figure 4d). It is also the first metabolite of carbamazepine in tomato plants and *Armoracia rusticana* root cultures [28,30]. Hence, the increase of succinic acid (DM_4) might affect the TCA cycle (tricarboxylic acid cycle) of *P. australis.*

Also, diclofenac and its hydroxylated metabolites were identified in *P. australis* extracts. It has been reported that formation of hydroxylated metabolites is the first step in the detoxification of diclofenac in the plant. These results reveal that P450 monooxygenases or peroxidases were involved to detoxify diclofenac [11].

In CBZ incubation, different transformation products were identified, which originated from different metabolism pathways (PCM). The 10,11-diOH pathway (PCM) has been investigated comprehensively in plants like cucumber, tomato, sweet potato, lettuce, carrot and horseradish [28,31]. The main transformed product is carbamazepine-10,11-epoxide (Martínez-Piernas et al., 2019), which has been identified in leaf, rhizome and root in both incubations levels 10 and 50 µM, as shown in Table S3 [32]. This is the first oxidation step, which was observed in different organisms from bacteria and fungi to mammals. It was conducted by cytochrome P450 and/or peroxidases, leading to different sub-pathways (PCM).

The first sub-pathway (PCM) started with cleavage of the epoxide bond and the hydroxylation to form 10,11-dihydro-10,11-dihydroxy-carbamazepine. This step is catalyzed by epoxide hydrolase enzymes [33]. A recent study reported further metabolism of 10,11-dihydro-10,11-dihydroxy-carbamazepine through hydroxylation of the benzene ring [32]. 10,11-dihydro-10,11-dihydroxy-carbamazepine was identified in root and rhizome extracts in both incubation concentrations, as shown in Table S3. However, 10,11-dihydro-10-hydroxy-carbamazepine was identified in leaf extracts in the 50 µM carbamazepine. It seems that the 10,11-dihydro-10,11-dihydroxy-carbamazepine was transferred to rhizome and leaf for further metabolism. This explains why it reaches the leaves in high concentration from the highly loaded rhizome tissue. Thus, *P. australis* can degrade carbamazepine through a 10,11-diOH pathway (PCM), initiated in the root and completed in leaf and rhizome.

Further, *P. australis* metabolized the carbamazepine-10,11-epoxide into 9-acridine carboxaldehyde, which is analogous to other studies [28,31,32]. The 9-acridine carboxaldehyde is a reactive compound that was identified in leaf, rhizome and root extracts of plants exposed to 10 and 50 µM carbamazepine. Further metabolites downstream from 9-acridine carboxaldehyde could not be detected. 9-acridine carboxaldehyde is typically transformed into acridone, which is a non-toxic compound with the formation of the two intermediates acridine and 9-hydroxy acridine [28]. In our conditions, they were below the detectable limit or did not arise. Because 9-acridine carboxaldehyde was identified in leaf, rhizome and root extracts in both concentrations, it may be assumed that there are further steps in the acridine metabolic pathway (PCM) that could be detected, like in lettuce, with longer incubation periods [32].

The last sub-pathway (PCM) is similar to the 10, 11-diOH pathway in the first steps. It includes a consecutive oxidation reaction on the aromatic group. Carbamazepine was metabolized to 2,3-dihydro-2,3-dihydroxy-carbamazepine which comprises two steps with the participation of a cytochrome P450 or a peroxidase and an epoxide hydrolase [33]. 2,3-dihydro-2,3-dihydroxy-carbamazepine was identified in the *P. australis* rhizome and root in both incubation levels. However, 2,3-dihydro-2,3-dihydroxy-carbamazepine was not detectable in leaf extracts. It is moreover possible that the leaf metabolizes CBZ to 4-hydroxy-carbamazepine as in the leaves of the tomato plant. It was identified formerly in lettuce after 4 days of exposure; however, the researchers spiked the lettuce with 1 mg L^−1^ carbamazepine [30,32].

Thus, it is possible that *P. australis* takes up and metabolizes carbamazepine in different mechanisms. Still, all the metabolites resemble phase I metabolism in plant metabolism and not the glycosylated transformation products (phase II) [3].

The mummichog algorithm of the DMF between control and incubated samples shows an induction in the eleven and twelve pathways due to incubation with DCF or CBZ, respectively. They were identified as glycolysis/gluconeogenesis, ascorbate and aldarate metabolism, fructose and mannose metabolism, galactose metabolism, the pentose phosphate pathway, arginine biosynthesis, alanine, aspartate and glutamate metabolism, purine metabolism, pyrimidine metabolism, glutathione metabolism and phenylalanine metabolism in DCF incubation.

After CBZ incubation, the pentose phosphate pathway, purine metabolism, pyrimidine metabolism, fatty acid biosynthesis, arachidonic acid metabolism, tyrosine metabolism, tryptophan metabolism, β-Alanine metabolism, arginine and proline metabolism, pantothenate and CoA biosynthesis, carbon fixation in photosynthetic organisms and folate biosynthesis pathways were induced after the incubation of CBZ.

The glucosinolate biosynthesis pathway provides the plant with defense compounds against biting insects and worms. Furthermore, the cytochrome P450 enzyme controls glucosinolate biosynthesis, which is involved in the metabolism of DCF and CBZ during phase I [11,34]. The decline in sugars after plant exposure to DCF and CBZ indicates an increase in energy consumption [35].

Pyrimidine metabolism could be proposed to be a result of glucosinolate biosynthesis pathway alteration, which up-regulates the biosynthesis of other stress-induced pathways [36]. Pyrimidine metabolism is enhanced and leads to products that could be used in the case of salvage, that is, recovery of infections and subsequent synthesis of secondary products with specific functions in defense mechanisms. Additionally, pyrimidine metabolism provides a source of β-alanine or β-aminobutyrate, which might be an important source for the pantothenate of coenzyme A [37].

Furthermore, fatty acid biosynthesis and the related pyruvate metabolism were induced after incubation of *P. australis* with DCF and CBZ. After incubation with DCF, *P. australis* exhibits some responses differently from incubation with CBZ, since it showed an increase in phenylalanine metabolism which aligned with the increase of quercetin content. The flavonoid 3′-monooxygenase and the flavonoid 3′,5′-hydroxylase enzymes responsible for the conversion of kaempferol to quercetin are cytochrome P450 plant types, which are triggered after incubation with DCF. However, the quercetin levels were lower after CBZ incubation (Figure 4d).

Glutathione metabolism was affected in DCF incubation but not under the influence of CBZ. Glutathione metabolism is a central part of the antioxidative ascorbate–glutathione cycle. Further, glutathione is critical for the detoxification of xenobiotics, environmental stress tolerance and, in the form of phytochelatins, also the retention of heavy metals [38]. The metabolism of DCF required Nicotinamide-Adenine Dinucleotide Phosphate (NADPH) as reductants [11]. Upon DCF incubation, succinic acid formed from the degradation of diclofenac might have fueled the TCA cycle, which has been detected significantly in extracts incubated with DCF (Figure 4c).

In 2019, Sivaram and coworkers reported a high impact on the TCA cycle in maize leaves exposed to pyrene [39]. Consequently, the γ-Aminobutyric acid (GABA) shunt bypasses two steps of the TCA cycle. Moreover, it has an important pathway under stress conditions and it is associated with numerous physiological responses, including the regulation of cytosolic pH, carbon fluxes into the TCA cycle, nitrogen metabolism, osmoregulation and plant-pathogen interaction. Increased GABA levels also occur in response to changing environmental conditions and represent another adaptive mechanism in the attempt to maintain the rate of respiration under certain harmful conditions (Araújo et al., 2012) [40].

The same result was observed in lettuce crops, which were exposed to different contaminants of emerging concern (CEC) concentrations [35]. Also, it was reported that aromatic hydrocarbons altered the osmotic balance in maize [39]. The alteration of previously mentioned biosynthetic pathways enhances the *P. australis* defense mechanisms. They also seem to be involved in the transformation of DCF and CBZ.

Finally, *P. australis* used the glutathione metabolism pathway to defend itself against the DCF and seemingly, used the unsaturated fatty acid pathway to protect itself during the incubation with CBZ. Hence, *P. australis* responded differently to the DCF and CBZ through changing its metabolic pathway regardless of the type of drug to some extent. Consequently, specific changes in several common metabolic pathways can be considered as a marker for pollutant exposure in *P. australis*. However, each drug has fingerprints of the alteration of distinct metabolic pathways, which might be connected to its metabolites and the enzymes involved in metabolism. Therefore, the induced or changed pathways could be used as indicators for the exposure of the plant to DCF or CBZ.

## 4. Materials and Methods

### 4.1. Reagents and Chemicals

LC-MS grade methanol and water were obtained from VWR, Darmstadt, Germany. Quercetin dehydrate (≥95%, high performance liquid chromatography (HPLC)) was purchased from Enzo Life Sciences GmbH, Lörrach, Germany. Diclofenac (>99%) was obtained from Cayman Chemical Company, Ann Arbor, Michigan, MI, USA. Glyphosate (100 quality level, HPLC), gabapentin (200 quality level, HPLC), monuron (100 quality level, HPLC), chloridazon (100 quality level, HPLC), carbetamide (100 quality level, HPLC), metobromuron (100 quality level, HPLC), sotalol (≥98%), quinoxyfen (100 quality level, HPLC), metconazol (100 quality level, HPLC) and fenofibrate (≥99%) were obtained from Sigma, Darmstadt, Germany. Metformin (300 quality level, HPLC) was obtained from Fluka, Buchs, Switzerland. Furthermore, chlorbromuron (99.24%) and diazinon (99.53%) were obtained from Dr. Ehrenstorfer, Augsburg, Germany. Carbamazepine, 2,3-dihydro-2,3-dihydroxycarbamazepine, 10,11-dihydro-10,11-dihydroxy-carbamazepine, 10,11-dihydro-10-hydroxy-carbamazepine, 9-acridine carboxaldehyde and carbamazepine-10,11-epoxide were kindly provided by the German Research Center for Environmental Health, Comparative Microbiome Analysis (COMI), Helmholtz Centrum of Munich, Munich, Germany.

### 4.2. Plant Samples

Twelve *P. australis* plants were grown in semi-hydroponic conditions in the greenhouse as described by Sauvêtre and Schröder earlier, in 2015 [7]. Plants were grown in Hoagland solution made of (in mg/L) 472.30 Ca(NO_3_)_2_·4H_2_O, 202.22 KNO_3_, 492.96 MgSO_4_·7H_2_O, 68.04 KH_2_PO_4_, 80.04 NH_4_NO_3_, H_3_BO_3_, 1.8 MnCl_2_·4H_2_O, 0.2 ZnSO_4_·7H_2_O, 0.1 CuSO_4_·5H_2_O, 0.025 NaMoO_4_ and 3.67 FeNa-Ethylenediaminetetraacetic acid. Plants (approximately 0.8 m in height) of uniform size were selected and placed into individual pots containing 2 L of spiked Hoagland solution Each pot contained one plant and was arranged in the greenhouse following a completely randomized design. The nutrient medium was spiked with a stock solution to reach the desired final concentration (diclofenac, 10 and 100 µM and carbamazepine, 10 and 50 µM, respectively). Control plants growing in Hoagland solution (spiked with the same amount of solvent as incubated plants) were used to obtain a reference plant matrix. Two pots were set up for each of the four exposure concentrations. Each assay consisted of duplicates arranged in the greenhouse following a randomized design. To compensate for water losses by evapo-transpiration, distilled water was added daily to the pots for a final volume of 2 L. Plants were exposed for 4 days before they were harvested. Harvested material was divided into roots, rhizomes and leaves, frozen in liquid nitrogen and stored at −81 °C until further processing. *P. australis* was kindly provided by the German Research Center for Environmental Health, Comparative Microbiome Analysis (COMI), Helmholtz Center Munich, Munich, Germany.

### 4.3. Extraction

Twelve samples of *P. australis* leaves, rhizomes and roots, respectively, were collected, frozen under liquid N2. Then, the samples were freeze-dried and milled (Retsch S1 planetary ball mill, Retsch GmbH, Haan, Germany). Duplicates of each plant part were extracted with (a) 100% methanol (MeOH), (b) 90% MeOH (MeOH-water-formic acid (FAC) (90:9.5:0.5, *v*/*v*/*v*), (c) MeOH-water (50:50, *v*/*v*) and (d) 100% water (H_2_O), respectively. The solvents containing 500 mg plant powder were sonicated (Sonorex super RK 106, Bandelin, Germany) for 10 min at 4 °C with 35 kHz frequency. Then, samples were centrifuged (Z 200 A Universal Compact Centrifuge, Hermle LaborTechnik GmbH, Wehingen, Germany) at 1500 rpm/261.6× *g* for 20 min and the supernatants were transferred to clean glass test tubes. The extraction process was triplicated in identical experimental conditions. Finally, the extracts were evaporated to dryness (using a SpeedVac, Fischer Scientific, Göteborg, Sweden) and dissolved in (50:50 (*v*/*v* %)) MeOH: H_2_O [18,26].

### 4.4. Instruments

Filtered samples and standards (with 22 µm filter, Analytics Shops, Munich, Germany) were separated by LC (Agilent 1260 Infinity) consisting of an autosampler, two columns, two binary pumps, an online degasser, a mixing chamber and a UV detector. The LC-system was used to perform reversed-phase and zwitterion hydrophilic interaction liquid chromatography (HILIC) in its serial coupling. The reversed-phase separation column was a Poroshell 120 EC-C18 (50.0 × 3.0 mm, 2.7 μm, Agilent Technologies Waldbronn, Germany). The HILIC column was a ZIC-HILIC column (150 × 2.1 mm, 5 μm, 200 Å, Merck Sequant, Umea, Sweden). Columns were coupled through a T-piece (Upchurch, IDEX Europe GmbH, Erlangen, Germany). The third port of the T-piece was connected to the HILIC flow pump. The injection volume was 10 μL. Further details, like the mobile phase of the RPLC-HILIC serial coupling and other settings, are described in References [24,25,26].

Samples and reference mixes were analyzed with a HRMS “time-of-flight” (TOF) mass spectrometer equipped with a Jet Stream ESI interface (Agilent Technologies, Waldbronn, Germany) and hyphenated with RPLC-HILIC chromatography. The parameters were as follows: 325 °C gas temperature, 10 L/min drying gas flow, 325 °C sheath gas temperature, 7.5 L/min sheath gas flow, 45-psi nebulizer operating pressure and 100 V fragmentor voltage. Ions were detected in positive ionization mode with a mass range of 50–2100 Daltons. The resolution of the instrument was better than 10,000 at *m/z* 922.

### 4.5. Quality Control of the RPLC-HILIC-ESI-TOF-MS System

The robustness and reproducibility of the RPLC-HILIC-ESI-TOF-MS system were tested with a standard mixture containing 13 different reference standards with a 20 µM final concentration. The mixture consisted of metformin, glyphosate, gabapentin, monuron, chloridazon, carbetamide, metobromuron, sotalol, chlorbromuron, diazinon, quinoxyfen, metconazol and fenofibrate. The mixture was injected at the beginning/end of the experiment series and at fixed intervals during the experiment. It was injected after each extraction batch.

The absolute variation between the literature monoisotopic mass and the mean of measured isotopic masses (Δppm) was computed according to the following equation:

Δppm = (monoisotopic mass of standard −mean of standard masses)/monoisotopic mass of standard × 10^6^ (1)

Moreover, the standard deviations (SD) of RT and Relative standard deviations (RSD) were calculated:

% of RSD = SD of compound RTs in different injection/Mean of compound RTs(2)

The results are summarized in (Tables S1 and S2). The absolute mass deviation ranged from 0.2 (Da) to 7 (Da). The RT standard deviation was less than 1%. Moreover, the relative standard deviation (%RSD) ranged from 0.5% to 3.6%. Thus, the results indicate the accuracy, repeatability and reproducibility of the LC system as previously reported in the investigation of plant metabolites in *Lemna minor* samples [18].

### 4.6. Data Evaluation

#### 4.6.1. Spectrometric Data Evaluation

Data acquired with MassHunter Workstation LC/MS Data Acquisition software B 05.00, (Agilent Technologies, Waldbronn, Germany) was subsequently analyzed with Profinder B.06.00 (Agilent Technologies, Waldbronn, Germany) to extract the so-called “features” by their retention times (RT), molecular mass and their peak intensity in various *P. australis* extracts. This was performed in a combination of the 3-fold injections of each sample after removing the features found in the corresponding blank samples. The parameters are set to a peak filter of 1000 counts peak height, ion species to “positive ions” with H^+^, Na^+^, K^+^ and NH4^+^, “charge state” to 1, the “expected RT” to ±3.00 min and the mass to ±10 ppm. The extracted ion chromatograms (EICs) were smoothed with a Gaussian function using 9 points function and 5000 points Gaussian width. This limits the result finally to 2000 compound groups.

#### 4.6.2. DCF and CBZ Transformation Products Detection

DCF and CBZ transformation product standards were analyzed using Agilent Profinder B.06.00 (Agilent Technologies). They were detected in the *P. australis* extracts, with masses estimated at ±10 ppm and RT ±0.3 min of the exact mass and RT of the standards, respectively. The metabolites were identified and suspected (when analytical standards were not available) after RPLC-HILIC-ESI-TOF-MS separation in the suspect analysis. (Suspects screening typically is performed with accurate and high-resolution mass spectrometers to observe the empirical formula of each molecule present and/or with tandem-mass spectrometry to observe specific fragment spectra). A local database was built using MassHunter PCDL Manager B.04.00 (Agilent Technologies, Waldbronn, Germany). Further, the logD (pH7) was the third parameter used to certify the identity of metabolites. The highly polar to polar compounds eluted from the HILIC column at RT < 15 min, with logD values below zero. The nonpolar compounds were eluted from the RP column at RT > 15 min, with logD values above zero. Metabolites within the criteria of mass, RT and logD (pH7) in the suspect analysis were considered.

#### 4.6.3. Statistical Data Analysis

Data statistical analyses were conducted with SIMCA 16 software (Malmö, Sweden). Further analysis and data evaluation were performed with Microsoft Access and Excel 2016 (Redmond, Washington, WA, USA) and OriginPro 2019, Origin Lab cooperation, Northampton, MA, USA.

The preprocessed data is a matrix. The rows are the exact masses, retention times (RTs) and abundances of each sample, which were listed in a Microsoft Excel Sheet. For statistical analysis, it is common to handle the data matrices with rows as observations and columns as compounds [36]. Therefore, the data was organized in the Microsoft Access Database file (DBF), which was exclusively built to be suitable for SIMCA 16. The main advantage of the data matrix is the inherent support to align quantitative data (plant part, plant number, extraction solvent and drug incubation) along with related metadata (i.e., feature annotations/abundance as columns and sample annotations as rows). In DBF, the RTs, masses and abundances were connected to the corresponding plant part (i.e., leaf, rhizome and root), plant number (i.e., plants 1 and 2), extraction solvent and drug incubation. Once the matrix was built, comprehensive statistical analyses could be performed by using the vast range of functions provided by the software. The matrix consisted of 432 observations (i.e., the incubation with/without DCF or CBZ) and 11,442 variables (features). Furthermore, the plant part, plant number and extraction solvent were used as secondary observations. The data was not transformed and centered; however, it was scaled. The data was analyzed according to the following two strategies, considering the statistical analyses in untargeted metabolomics. Further information about the data setup can be found in the supplementary material. By default, SIMCA provides an algorithm called “cross-validation” to get the most valid model by calculating the adequate number of principal components to prevent overfitting of the data in the model. Furthermore, the software provides a large number of visual diagrams (score plots (with Hotelling’s ellipse), DModX (Distant to Model) and statistic tables to assess the quality of the model in addition to the R^2^ and Q^2^.

1. Metabolite fingerprinting was used to capture metabolite patterns across metabolite profiles. They are characterized without further identification steps (i.e., without need for standard reference material). Partial Least Squares (PLS) and Orthogonal Partial Least Squares regression-Discriminant Analysis (OPLS-DA) were used to relate sets of X-variables (such as plant part, plant number, extraction solvent and drug incubation) to the metabolites matrix. SIMCA 16 has a tool called Multiblock Orthogonal Component Analysis (MOCA). MOCA’s concept is used to accomplish a fast and accurate analysis of multiple blocks of data (variables) registered for the same set of observations. MOCA aims at extracting the information in complex multi-block data analytics. Furthermore, it will extract two sets of components: the joint and the unique components. The quality of the models is described by R^2^ and Q^2^ values, where R^2^ is the proportion of variance in the data explained by the models and indicates the goodness of fit and Q^2^ is the proportion of variance in the data predictable by the model and expresses predictability [41].
2. Metabolite profiling which uses sets of predefined metabolites were studied in different samples of *P. australis* and differences in metabolites were usually related to the incubation with DCF or CBZ. Metabolite/variable selection was conducted to observe only the most significant metabolite candidates that explain the differences between the samples using S- and contribution-plots. The statistical models were built with confidence limits at 95%. Also, the differentiating metabolic profile (DMF) was chosen based on their contribution to the variation and correlation within the data sets. The related metabolic pathways were analyzed using MetaboAnalyst 4.0. Moreover, their contributions and biological clarifications were described based on the Kyoto Encyclopedia of Genes and Genomes (KEGG) database. The KEGG pathway analysis tool was used by the Arabidopsis thaliana database. The pathway analysis module combines the enrichment analysis and topology analysis based on KEGG. Fisher’s test was used to generate *p* values. The *p* value was equal to 0.05, which indicates the fundamental connection of the identified metabolite with their respective metabolite and not due to the random chance [42,43].

## 5. Conclusions

Metabolites of *P. australis* influenced by pharmaceuticals were investigated using RPLC-HILIC-ESI-TOF-MS. The experimental data and the statistical analysis revealed a change in the metabolites’ fingerprint between the different extracts, different plant parts and upon incubation with DCF and CBZ. The PLS and OPLS-DA identified the statistically significant clusters between the different groups. Further, significant DMF was determined in *P. australis* after the incubation with DCF or CBZ, individually. Different metabolic pathways were predicted from the statically identified DMF. These pathways were related mainly to the defense of the plant against stressful environmental conditions. *P. australis* adapted to each drug differently. *P. australis* could putatively use the glutathione metabolism pathway and unsaturated fatty acid pathway to protect itself during the incubation with DCF and CBZ, respectively. The results reveal insights into the metabolic profile of the species´ adaptation to different pollutants. This study may set a cornerstone for understanding the changes in the plant metabolism after incubation with DCF and CBZ. Also, the mass spectrometric untargeted metabolomics strategy has a substantial role in investigating the biochemical changes and metabolic adaptation of plants in xenobiotics exposure cases.

## Figures and Tables

**Table 1:** List of diclofenac (DCF) transformation products detected in different *Phragmites australis* leaf, rhizome and roots incubated with 10 and 100 µM diclofenac samples with the monoisotopic mass in the literature (L), the mean monoisotopic mass of *Phragmites australis* (Ph), the variation between them, mean RT of *Phragmites australis* (Ph), standard deviation, relative standard deviation and LogD (pH = 7.4) were listed. The logD values were predicted from ChemAxon software (https://disco.chemaxon.com/apps/demos/logd/).

DCF Transformed Products	Name	Mono Isotopic Mass (Da) (L) (Rajab, Greco et al. 2013)	Mean Mono Isotopic Mass (Da) (Ph)	Δppm	Mean RT (Min) (Ph)	SD of RT (Min)	RSD	LogD (pH = 7.4)	Leaf	Rhizome	Root
**DM_1**	2-Hydroxypropanoic acid	152.0473	152.0475	−0.99	8.1	0.05	0.67	−1.86	√	√	√
**DM_2**	2-(Hydroxymethyl)benzene-1,4-diol	140.0473	140.0472	0.71	12.5	0.06	0.51	0.60	√	√	√
**DM_3**	2-Hydroxysuccinic acid	134.0215	134.0215	0.07	12.6	0.03	0.23	−6.81	√	√	√
**DM_4**	Succinic acid	118.0266	118.0271	−3.95	6.8	0.04	0.55	−1.99	√	√	√
**DM_5**	Fumaric acid	116.0101	116.01	0.49	12.6	0.05	0.42	−2.00	√	√	√
**DM_6**	Propane-1,2,3-triol	92.0473	92.04703	2.9	7.2	0.07	0.99	−1.84	√	×	×
**DM_7**	2-Hydroxypropanoic acid	90.0317	90.03207	−4.07	12.1	0.04	0.32	−1.00	√	×	×

## Data Availability

The data is available at https://www.ebi.ac.uk/metabolights/ with ID—sMTBLS2321.

## Supplementary Materials

The supplementary materials are available online at https://www.mdpi.com/2218-1989/11/1/2/s1, Figure S1: Retention time (RT)/Mass plot of the background was analyzed by RPLC-HILIC-ESI-TOF-MS in positive electrospray ionization mode, Figure S2: The Q^2^/ R^2^ Overview plot displays the individual cumulative R^2^ (green columns) and Q^2^ (blue columns) and Q^2^ for the goodness of fits and cross-validation parameters (a) *P. australis* different parts. (b) *P. australis* different incubation, Figure S3: EICs were corresponding to measured diclofenac (right) and the reference standard (left), which were identified in the extracts of *Phragmites australis* leaf, rhizome, and roots incubated with 10 and 100 μM diclofenac. Also, EICs relative to transformed products are suspected in the extracts of *Phragmites australis* leaf, rhizome, and roots incubated with 10 and 100 μM diclofenac, Figure S4: EICs were corresponding to carbamazepine (CBZ) and its transformed product standards (left), which were identified in the extracts of *Phragmites australis* leaf, rhizome, and roots incubated with 10 and 50μM carbamazepine (measured right), Table S1: The standards compounds of the quality control external calibration mixture, monoisotopic mass in the literature (L), monoisotopic in different injection and the mean of them, the variation between monoisotopic mass in the literature (L), and the mean of measured monoisotopic mass, and mean mass standard deviation (SD) are listed, Table S2: The standards compounds of the quality control external calibration mixture, the single RT, mean RT of the different injections, mean RT standard deviation (SD), and relative standard deviation (RSD) of the standards are listed, Table S3: List of carbamazepine (CBZ) transformed product identified in *Phragmites australis* different samples with the mean monoisotopic mass in the standards (S), the mean monoisotopic mass of *Phragmites australis* (Ph), the variation between them, mean RT of standards (S), mean RT of *Phragmites australis* (Ph), and the variation between them were listed, Table S4: The differentiating metabolic profile (DMF) metabolites of *Phragmites australis* due to incubation with 10 & 100 µM diclofenac were extracted from the S-plot, monoisotopic mass, *p*-value, and t. score are listed, Table S5. The differentiating metabolic profile (DMF) metabolites of *Phragmites australis* due to incubation with 10 & 50 µM carbamazepine were extracted from the S-plot, monoisotopic mass, *p*-value, and t.score are listed.

## References

[1] Tohge T., Nishiyama Y., Hirai M.Y., Yano M., Nakajima J.-I., Awazuhara M., Inoue E., Takahashi H., Goodenowe D.B., Kitayama M. (2007). Identification of Genes Involved in Anthocyanin Accumulation by Integrated Analysis of Metabolome and Transcriptome in Pap1-Overexpressing Arabidopsis Plants.

[2] Sandermann H., Diesperger H., Scheel D. (1977). Metabolism of Xenobiotics by Plant Cell Cultures.

[3] Schröder P., Scheer P., Belford E.J.D. (2002). Metabolism of organic xenobiotics in plants: Conjugating enzymes and metabolic endpoints. Minerva Biotecnol..

[4] Schröder P., Maier H., Debus R. (2005). Detoxification of Herbicides in *Phragmites australis*. Z. Nat. C.

[5] Villette C., Maurer L., Wanko A., Heintz D. (2019). Xenobiotics metabolization in Salix alba leaves uncovered by mass spectrometry imaging. Metabolomics.

[6] Letzel M., Metzner G., Letzel T. (2009). Exposure assessment of the pharmaceutical diclofenac based on long-term measurements of the aquatic input. Environ. Int..

[7] Sauvêtre A., Schröder P. (2015). Uptake of carbamazepine by rhizomes and endophytic bacteria of *Phragmites australis*. Front. Plant Sci..

[8] Thelusmond J.-R., Kawka E., Strathmann T.J., Cupples A.M. (2018). Diclofenac, carbamazepine and triclocarban biodegradation in agricultural soils and the microorganisms and metabolic pathways affected. Sci. Total Environ..

[9] Vieno N., Sillanpää M. (2014). Fate of diclofenac in municipal wastewater treatment plant—A review. Environ. Int..

[10] Hai F.I., Yang S., Asif M.B., Sencadas V., Shawkat S., Sanderson-Smith M., Gorman J., Xu Z.-Q., Yamamoto K. (2018). Carbamazepine as a Possible Anthropogenic Marker in Water: Occurrences, Toxicological Effects, Regulations and Removal by Wastewater Treatment Technologies. Water.

[11] Huber C., Bartha B., Schröder P. (2012). Metabolism of diclofenac in plants—hydroxylation is followed by glucose conjugation. J. Hazard. Mater..

[12] Park M.G., Blossey B. (2008). Importance of plant traits, and herbivory for invasiveness of *Phragmites australis* (Poaceae). Am. J. Bot..

[13] Gray K.R., Biddlestone A.J. (1995). Engineered reed-bed systems for wastewater treatment. Trends Biotechnol..

[14] Zhou B., Xiao J.F., Tuli L., Ressom H.W. (2012). LC-MS-based metabolomics. Mol. Biosyst..

[15] Beale D., Pinu F., Kouremenos K., Poojary M., Narayana V., Boughton B., Kanojia K., Dayalan S., Jones O., Dias D. (2018). Review of recent developments in GC–MS approaches to metabolomics-based research. Metabolomics.

[16] Emwas A.-H., Roy R., McKay R.T., Tenori L., Saccenti E., Gowda G.A.N., Raftery D., Alahmari F., Jaremko L., Jaremko M. (2019). NMR Spectroscopy for Metabolomics Research. Metabolites.

[17] Aretz I., Meierhofer D. (2016). Advantages, and pitfalls of mass spectrometry-based metabolome profiling in systems biology. Int. J. Mol. Sci..

[18] Wahman R., Grassmann J., Schröder P., Letzel T. (2019). Plant metabolomic workflows using reversed-phase LC and HILIC with ESI-TOF-MS. LCGC N. Am..

[19] Riach A.C., Perera M.V.L., Florance H.V., Penfield S.D., Hill J.K. (2015). Analysis of plant leaf metabolites reveals no common response to insect herbivory by *Pieris rapae* in three related host-plant species. J. Exp. Bot..

[20] Gromski P.S., Xu Y., Kotze H.L., Correa E., Ellis D.I., Armitage E.G., Turner M.L., Goodacre R. (2014). Influence of missing values substitutes on multivariate analysis of metabolomics data. Metabolites.

[21] Lamichhane S., Sen P., Dickens A.M., Hyötyläinen T., Orešič M. (2018). Chapter Fourteen—An Overview of metabolomics data analysis: Current tools and future perspectives. Compr. Anal. Chem..

[22] Prinsloo G., Vervoort J. (2018). Identifying anti-HSV compounds from unrelated plants using NMR and LC-MS metabolomic analysis. Metabolomics.

[23] Worley B., Powers R. (2013). Multivariate Analysis in Metabolomics. Curr. Metab..

[24] Bieber S., Greco G., Grosse S., Letzel T. (2017). RPLC-HILIC, and SFC with mass spectrometry: Polarity-extended organic molecule screening in environmental (Water) samples. Anal. Chem..

[25] Greco G., Grosse S., Letzel T. (2013). Serial coupling of reversed-phase and zwitterionic hydrophilic interaction LC/MS for the analysis of polar and nonpolar phenols in wine. J. Sep. Sci..

[26] Wahman R., Graßmann J., Sauvêtre A., Schröder P., Letzel T. (2020). *Lemna minor* studies under various storage periods using extended-polarity extraction and metabolite non-target screening analysis. J. Pharm. Biomed. Anal..

[27] Rajab M., Greco G., Heim C., Helmreich B., Letzel T. (2013). Serial coupling of RP and zwitterionic hydrophilic interaction LC-MS: Suspects screening of diclofenac transformation products by oxidation with a boron-doped diamond electrode. J. Sep. Sci..

[28] Sauvetre A., May R., Harpaintner R., Poschenrieder C., Schröder P. (2018). Metabolism of carbamazepine in plant roots and endophytic rhizobacteria isolated from *Phragmites australis*. J. Hazard. Mater..

[29] Hess D. (1975). Plant Physiology.

[30] Riemenschneider C., Seiwert B., Schwarz D., Reemtsma T. (2017). Extensive Transformation of the pharmaceutical carbamazepine following uptake into intact tomato plants. Environ. Sci. Technol..

[31] Riemenschneider C., Seiwert B., Goldstein M., Al-Raggad M., Salameh E., Chefetz B., Reemtsma T. (2017). An LC-MS/MS method for the determination of 28 polar environmental contaminants and metabolites in vegetables irrigated with treated municipal wastewater. Anal. Methods.

[32] Martínez-Piernas A.B., Nahim-Granados S., Polo-López M.I., Fernández-Ibáñez P., Murgolo S., Mascolo G., Agüera A. (2019). Identification of transformation products of carbamazepine in lettuce crops irrigated with Ultraviolet-C treated water. Environ. Pollut..

[33] Tybring G., von Bahr C., Bertilsson L., Collste H., Glaumann H., Solbrand M. (1981). Metabolism of carbamazepine and its epoxide metabolite in human and rat liver in vitro. Drug Metab. Dispos..

[34] Lee D.-K., Ahn S., Cho H.Y., Yun H.Y., Park J.H., Lim J., Lee J., Kwon S.W. (2016). Metabolic response induced by parasitic plant-fungus interactions hinder amino sugar and nucleotide sugar metabolism in the host. Sci. Rep..

[35] Hurtado C., Parastar H., Matamoros V., Piña B., Tauler R., Bayona J.M. (2017). Linking the morphological and metabolomic response of *Lactuca sativa* L exposed to emerging contaminants using GC × GC-MS and chemometric tools. Sci. Rep..

[36] Tantikanjana T., Mikkelsen M.D., Hussain M., Halkier B.A., Sundaresan V. (2004). Functional Analysis of the tandem-duplicated P450 genes SPS/BUS/CYP79F1 and CYP79F2 in glucosinolate biosynthesis and plant development by Ds transposition-generated double mutants. Plant Physiol..

[37] Zrenner R., Stitt M., Sonnewald U., Boldt R. (2006). Pyrimidine and purine biosynthesis and degradation in plants. Annu. Rev. Plant Biol..

[38] Gong B., Sun S., Yan Y., Jing X., Shi Q. (2018). Glutathione Metabolism and Its Function in Higher Plants Adapting to Stress. Antioxidants and Antioxidant Enzymes in Higher Plants.

[39] Sivaram A.K., Subashchandrabose S.R., Logeshwaran P., Lockington R., Naidu R., Megharaj M. (2019). Metabolomics reveals defensive mechanisms adapted by maize on exposure to high molecular weight polycyclic aromatic hydrocarbons. Chemosphere.

[40] Araújo W.L., Nunes-Nesi A., Nikoloski Z., Sweetlove L.J., Fernie A.R. (2012). Metabolic control and regulation of the tricarboxylic acid cycle in photosynthetic and heterotrophic plant tissues. Plant Cell Environ..

[41] Löfstedt T., Trygg J. (2011). OnPLS—A novel multiblock method for the modeling of predictive and orthogonal variation. J. Chemom..

[42] Chong J., Wishart D.S., Xia J. (2019). Using MetaboAnalyst 4.0 for Comprehensive and Integrative Metabolomics Data Analysis. Curr. Protoc. Bioinform..

[43] Chong J., Yamamoto M., Xia J. (2019). MetaboAnalystR 2.0: From Raw Spectra to Biological Insights. Metabolites.

