# Supplementary material for: Untargeted Metabolomics Studies on Drug-Incubated Phragmites australis Profiles
Source: Metabolites. 2020 Dec 22;11(1):2. doi: 10.3390/metabo11010002 (PMC7822174; doi:10.3390/metabo11010002)
Supplement: Supplementary file 1 [file metabolites-11-00002-s001.pdf]

Untargeted metabolomics studies on drug-incubated Phragmites australis profiles

Rofida Wahman[1], Andres Sauvêtre [2+], Peter Schröder [2], Stefan Moser [3], Thomas Letzel[1,4,*]

[1] Chair of Urban Water Systems Engineering, Technical University of Munich, Am Coulombwall 3, 85748 Garching, Germany.

[2] German Research Center for Environmental Health, Research Unit Comparative Microbiome Analysis, Helmholtz Centrum Munich, Ingolstadt street 1, 85764 Neuherberg, Germany

[3] Stefan Moser Process Optimization, Weberweg 3, D-83131 Nußdorf am Inn, Germany

[4] Analytisches Forschungsinstitut für Untarget Screening GmbH (AFIN-TS), Am Mittleren Moos 48, D - 86167 Augsburg, Germany

*Correspondence: T.letzel@tum.de

+ Current address: HydroSciences Montpellier, UMR 5569, Faculté de Pharmacie, University of Montpellier, Avenue Charles Flahault 15, 34000, Montpellier, France

2. Materials and methods:

2.1. Data Processing:

2.1.1. Access data file Creation

The experiment setup was inserted into the access database. Each run has a unique numerical ID from 1 to 432. Masses, RTs, and an abundance of compounds from each run were merged to the ID by inserting them into the access database. The internal Access programming helped to visualization and check that all the data was correctly inserted. This was done by reviewing the graphs and the corresponding pre documented additional data like (Solvent, part of the plant …..). After this from the dataset, a pivot matrix data table was calculated.

The pivot data table was arranged that masses@Rt are used as rows while the columns documented the accompanied abundance. The algorithm to attach the abundance to mass@Rt used the first value of abundance. Different approaches of mapping were tested such as (Mean, Average, First Value, Last Value…) but turned out to process a similar outcome in further analysis. Therefore, combination mass@Rt has occurred only in a unique combination with abundance.

Because of the limited possible number of columns (IDs) in Access the data table needed to be split into single excel files via a script before reunion it in SIMCA.

After the Excel, files with all the runs were merged in SIMCA the data table needed to be transposed to treat the different mass@RT as variables (Columns) and the different runs as observations (Rows).

After the union and transposing the data, the additional documentation like (Solvent, part of the plant …..) was pasted into SIMCA as well.

In the statistical software (SIMCA), the additional information (Solvent, part of the plant …..) were defined as secondary observations. This means that this information is not used within the developed models as variables.

As expected, some variables Mass@RT weren't found in all observation runs. Accordingly, the pivot table used to merge the data did document this with missing data. This is not very helpful in analyzing the data because the statistical software would see this data as "missing". Instead of "no occurrence". To put this right the empty cells were replaced with zeros

After this, the data was used to build the first model. In this starting PCA analysis, the untreated data was stored as a reference and to start the basic analysis with further models.

This is an especially important step to get an overview of the overall pattern in the data. The most important tools are a.) the score scatters plot which presents the consistency of the data using the uses the hosteling ellipse. This ellipse represents a 95% confidence interval in the multidimensional space. Observation outside this ellipse is remarkably interesting and needs to be investigated. Sometimes these Observations could also be identified as outliers.

Also the DModX "Distance to the Model in X" could give insights about the portion of the Variance (Predicted – Observed) which couldn't be described by the Model, to get a better understanding of what the model is capable of and what might be very unlikely and need more detailed investigation.

In the data, no anomalies or outliers have been found.

Within the first analysis, the underlying correlation pattern is represented with clusters in the score scattered plot, who summarized the information of all investigation runs in each one data point. With the help of the 2nd observations, the data set can be colored accordingly to check if the for-instance solvent or part of the plant does have significant uniqueness to expose the observation in one of the clusters. This is an easy way to analyze the clusters using the secondary information of documentation without considering the extra information to build the model. Then, the OPLS-DA model was built as illustrated in the results part.

2.1.2. Metabolomics data analysis

The DMF of *Phragmites australis* assigned with the OPLS-DA and S-plots were extracted. The extracted data were returned to the original data. It is impossible to identify a pathway depend on just a mass. To get around this issue, a key concept is to shift the unit of analysis from individual compounds to individual pathways or a group of functionally related compounds. The mummichog algorithm is the first implementation of this concept to infer pathway activities from a ranked list of MS peaks identified by untarget metabolomics. The original algorithm implements an over-representation analysis (ORA) method to evaluate pathway-level enrichment based on significant features assigned with the statistical analysis. Users need to specify a pre-defined cutoff based on *p*-values. For further details about the original implementation, please refer to Li *et al.* 2013. The mass accuracy was set to 5 ppm on the positive mode. The *p*-value cutoff was assigned to 0.05 to delineate between significantly enriched and non-significantly enriched features.

Table S1. The standards compounds of the quality control external calibration mixture, monoisotopic mass in the literature (L), monoisotopic in different injection and the mean of them, the variation between monoisotopic mass in the literature (L), and the mean of measured monoisotopic mass, and mean mass standard deviation (SD) are listed.

Name	Mono isotopic Mass (Da) (L)	Mono isotopic Mass (Da) (1st inj.)	Mono isotopic Mass (Da) (2nd inj.)	Mono isotopic Mass (Da) (3rd inj.)	Mono isotopic Mass (Da) (4th inj.)	Mono isotopic Mass (Da) (5th inj.)	Mono isotopic Mass (Da) (6th inj.)	Mean Mono isotopic Mass (Da)	Δ ppm	SD
**Metformin**	129.1014	129.1006	129.1001	129.1000	129.1005	129.1015	129.1014	129.1007	5.78	0.001
**Glyphosat**	169.0140	169.0132	169.0134	169.0138	169.0123	169.0120	169.0136	169.0131	5.62	0.001
**Gabapentin**	171.1259	171.1252	171.1254	171.1254	171.1231	171.1231	171.1253	171.1246	7.81	0.001
**Monuron**	198.0560	198.0566	198.0568	198.0569	198.0563	198.0564	198.0564	198.0566	-2.96	0
**Chloridazon**	221.0356	221.0359	221.0362	221.0361	221.0355	221.0359	221.0355	221.0359	-1.22	0
**Carbetamid**	236.1161	236.1176	236.1179	236.1180	236.1164	236.1169	236.1169	236.1173	-5.1	0.001
**Metobromuron**	258.0004	258.0012	258.0014	258.0010	258.0006	258.0025	258.0060	258.0021	-6.54	0.002
**Sotalol**	272.1195	272.1193	272.1193	272.1192	272.1192	272.1211	272.1195	272.1196	-0.55	0.001
**Chlorbromuron**	291.9615	291.9616	291.9614	291.9613	291.9613	291.9614	291.9614	291.9614	0.21	0
**Diazinon**	304.1010	304.1010	304.1019	304.1014	304.1004	304.1001	304.1022	304.1012	-0.42	0.001
**Quinoxyfen**	306.9967	306.9961	306.9961	306.9976	306.9948	306.9964	306.9967	306.9963	1.32	0.001
**Metconazol**	319.1451	319.1456	319.1452	319.1443	319.1452	319.1462	319.1451	319.1453	-0.46	0.001
**Fenofibrat**	360.1128	360.1135	360.1141	360.1137	360.1132	360.1141	360.1139	360.1138	-2.58	0

Table S2. The standards compounds of the quality control external calibration mixture, the single RT, mean RT of the different injections, mean RT standard deviation (SD), and relative standard deviation (RSD) of the standards are listed.

Name	RT (Min) (1st inj.)	RT (Min) (2ndinj.)	RT (Min) (3rdinj.)	RT (Min) (4thinj.)	RT (Min) (5th inj.)	RT (Min) (6thinj.)	Mean RT (Min)	SD	% RSD
**Metformin**	14.33	14.27	14.07	14.30	14.30	14.27	14.26	0.09	0.7
**Glyphosat**	13.73	13.72	14.39	13.85	14.03	13.81	13.92	0.25	1.8
**Gabapentin**	7.64	7.52	7.48	7.28	7.85	7.58	7.56	0.19	2.5
**Monuron**	24.32	24.22	24.25	24.59	24.59	24.23	24.37	0.17	0.7
**Chloridazon**	22.00	21.93	21.99	21.45	21.36	21.85	21.76	0.28	1.3
**Carbetamid**	23.90	23.84	23.84	22.66	22.65	23.85	23.46	0.62	2.6
**Metobromuron**	26.27	26.16	26.21	25.67	25.92	26.05	26.04	0.22	0.9
**Sotalol**	9.60	9.23	9.11	9.19	9.80	9.90	9.47	0.34	3.6
**Chlorbromuron**	26.92	27.61	27.66	27.41	27.72	28.00	27.55	0.37	1.3
**Diazinon**	33.45	33.49	33.44	33.07	33.38	34.33	33.53	0.42	1.3
**Quinoxyfen**	35.17	35.54	35.20	35.17	34.52	34.52	35.02	0.41	1.2
**Metconazol**	28.64	28.59	28.57	28.65	28.49	28.28	28.54	0.14	0.5
**Fenofibrat**	32.90	33.11	32.87	33.16	33.19	33.38	33.10	0.19	0.6

Table S3. List of carbamazepine (CBZ) transformed product identified in *Phragmites australis* different samples with the mean monoisotopic mass in the standards (S), the mean monoisotopic mass of *Phragmites australis* (Ph), the variation between them, mean RT of standards (S), mean RT of *Phragmites australis* (Ph), and the variation between them were listed. The logD values were predicated from ChemAxon software (https://disco.chemaxon.com/apps/demos/logd/)

CBZ Transformed Products	Mean Mono isotopic Mass (Da) (S)	Mean Mono isotopic Mass (Da) (Ph)	Δ ppm	Mean RT (Min) (S)	Mean RT (Min) (Ph)	Δ RT	LogD (pH=7.4)
**Carbamazepine-10,11-epoxide**	252.0903	252.0891	4.81	23.57	23.39	0.18	1.97
**10,11-Dihydro-10,11-dihydroxy-carbamazepine**	270.10	270.0994	2.16	22.34	22.12	0.22	0.81
**10,11-Dihydro-10-hydroxy-carbamazepine**	254.1055	254.1044	4.33	23.06	22.93	0.13	1.73
**9-Acridine carboxaldehyde**	207.0684	207.0682	1.10	33.46	33.27	-0.19	2.98
**2,3-Dihydro-2,3-dihydroxy-carbamazepine**	270.1	270.0994	2.16	5.37	5.20	0.17	-0.13

Table S4. The differentiating metabolic profile (DMF) metabolites of *Phragmites australis* due to incubation with 10 & 100 µM diclofenac were extracted from the S-plot, monoisotopic mass, *p*-value, and t. score are listed

Monoisotopic mass	*p*-value	t.score	Monoisotopic mass	*p*-value	t.score
68.0234	0.030106	0.23683	155.0692	0.015114	0.237591
68.0264	0.022125	0.166365	156.006	0.068255	0.18542
69.0214	0.039998	0.289065	159.0894	0.021152	0.171257
69.0577	0.019497	0.164378	162.0533	0.021843	0.153318
69.0586	0.024319	0.219215	170.0186	0.024278	0.200248
74.034	0.017752	0.260474	180.0193	0.015685	0.262302
78.9832	0.032256	0.307147	180.021	0.029023	0.236323
84.0216	0.041884	0.125878	180.021	0.022126	0.224066
84.0216	0.068541	0.213997	180.0215	0.027404	0.222718
84.0216	0.021316	0.091268	180.0215	0.01918	0.170941
84.0219	0.030039	0.250664	194.115	0.021004	0.155939
87.23	0.095546	0.279915	197.0906	0.032444	0.17429
87.23	0.028594	0.151951	197.0906	0.029217	0.160687
89.0477	0.030512	0.191343	202.1285	0.023499	0.194255
89.0477	0.022961	0.212673	208.9804	0.033769	0.18385
97.9683	0.027681	0.239861	208.9804	0.026954	0.166529
101.05	0.020528	0.258464	214.0096	0.015891	0.198022
103.9845	0.037352	0.18452	214.0096	0.017781	0.175736
103.9848	0.021889	0.127428	219.1034	0.016703	0.183509
104.0351	0.022192	0.200123	240.1469	0.022109	0.147296
105.0393	0.025857	0.213747	241.1054	0.018804	0.27248
105.0429	0.05	0.259898	255.0755	0.029164	0.126878
105.0429	0.019939	0.121926	255.1678	0.020379	0.150431
105.0776	0.022807	0.233001	255.1678	0.040065	0.227302
105.0788	0.027732	0.20118	270.0712	0.059532	0.185823
110.0845	0.026338	0.168961	270.0723	0.056079	0.178862
115.0287	0.022279	0.189553	270.9991	0.019459	0.2047
115.0635	0.050546	0.199762	273.1539	0.052786	0.207747
117.0782	0.042451	0.154643	273.156	0.030913	0.140906
117.0782	0.035544	0.124902	273.156	0.037379	0.114409
117.0782	0.06642	0.192607	273.156	0.022518	0.080971
117.0792	0.034949	0.149076	273.1788	0.015009	0.205866
117.1149	0.020489	0.152183	276.0773	0.030484	0.140702
118.0413	0.022657	0.106052	282.1656	0.016142	0.259492
120.0435	0.039739	0.206169	303.1539	0.032277	0.224316
120.0435	0.078967	0.351932	303.1546	0.044533	0.23808
120.0436	0.021475	0.203871	308.1591	0.032854	0.187574
120.0436	0.016406	0.226417	320.0991	0.016778	0.204282
126.0411	0.023179	0.132794	320.0991	0.013498	0.240874
129.0783	0.018318	0.188127	320.0991	0.016778	0.204282
131.0947	0.027175	0.102577	320.0991	0.013498	0.240874
131.0951	0.045885	0.118508	368.1104	0.030716	0.121481
131.0951	0.022295	0.075155	368.1104	0.030716	0.121481
137.0814	0.025135	0.148162	373.1014	0.021469	0.268151
144.0367	0.079941	0.309844	373.1014	0.021469	0.268151
144.0379	0.018853	0.13714	532.6444	0.034617	0.174168
144.0391	0.023973	0.101013	532.6444	0.034617	0.174168
147.9867	0.025314	0.205985	607.2324	0.043548	0.207635
155.0686	0.016005	0.182058	607.2324	0.043548	0.207635

Table S5. The differentiating metabolic profile (DMF) metabolites of *Phragmites australis* due to incubation with 10 & 50 µM carbamazepine were extracted from the S-plot, monoisotopic mass, *p*-value, and t.score are listed

Monoisotopic mass	*p*-value	t.score	Monoisotopic mass	*p*-value	t.score
70.0536	0.013284	0.194421	186.1002	0.023673	0.249651
79.9409	0.061161	0.164513	190.1096	0.025954	0.214713
83.0374	0.010699	0.146955	201.1732	0.015503	0.10034
84.0215	0.061734	0.23701	206.1515	0.006323	0.136052
87.0323	0.059627	0.204729	215.1882	0.004611	0.212767
89.0479	0.01228	0.145846	216.0968	0.00502	0.173375
89.048	0.00947	0.189974	217.9675	0.002192	0.167609
89.0482	0.050044	0.23896	219.1104	0.067305	0.167342
103.0993	0.023768	0.123503	227.0906	0.012969	0.208512
103.9847	0.017263	0.148329	227.0907	0.076145	0.287427
103.9849	0.045547	0.162259	227.1889	0.004641	0.197283
103.985	0.006327	0.238841	241.2396	0.029797	0.211693
103.9851	0.056276	0.212792	241.2767	0.050552	0.292318
105.0423	0.08038	0.287973	244.1779	0.019101	0.336869
105.0427	0.08038	0.287973	249.1216	0.026224	0.27147
105.0787	0.007499	0.275313	251.0576	0.023621	0.238833
111.0433	0.029081	0.144429	255.0234	0.020722	0.247003
113.0855	0.035166	0.161382	287.2807	0.026274	0.346846
114.9497	0.019858	0.235099	289.0658	0.038021	0.151319
115.0266	0.03549	0.149447	294.0162	0.017712	0.346654
115.0266	0.038415	0.171592	295.2506	0.020323	0.310185
115.0633	0.022501	0.18596	301.1896	0.048709	0.278219
117.0785	0.041413	0.328885	302.1923	0.02723	0.325911
119.9588	0.033128	0.180568	303.2487	0.024313	0.397787
119.9589	0.007138	0.132	330.075	0.020419	0.266759
120.0436	0.037921	0.199761	330.1892	0.037219	0.230546
120.0436	0.03734	0.156266	343.2363	0.023681	0.241985
120.0436	0.034371	0.19167	344.1459	0.017875	0.301713
120.0436	0.037921	0.199761	352.0677	0.031794	0.327461
125.0143	0.0077	0.214742	364.0989	0.035662	0.201401
126.0654	0.008069	0.204016	382.1076	0.048453	0.203364
129.0425	0.053529	0.362021	382.147	0.059465	0.191069
129.0426	0.053529	0.362021	384.1757	0.053612	0.161827
137.0783	0.019625	0.203304	384.1787	0.062787	0.154608
138.9917	0.008553	0.133195	384.1787	0.066648	0.157788
147.9796	0.024155	0.218384	416.2029	0.023989	0.224429
147.9796	0.026522	0.232374	419.2163	0.030838	0.288205
147.9854	0.024155	0.218384	479.4187	0.020506	0.260596
157.1463	0.008491	0.21146	481.4001	0.026541	0.331124
167.9698	0.009256	0.132112	512.1178	0.030593	0.267982
171.1086	0.007084	0.196078	606.9501	0.05694	0.262249
180.0167	0.021764	0.191732	869.6639	0.069832	0.207301
186.099	0.023673	0.249651			

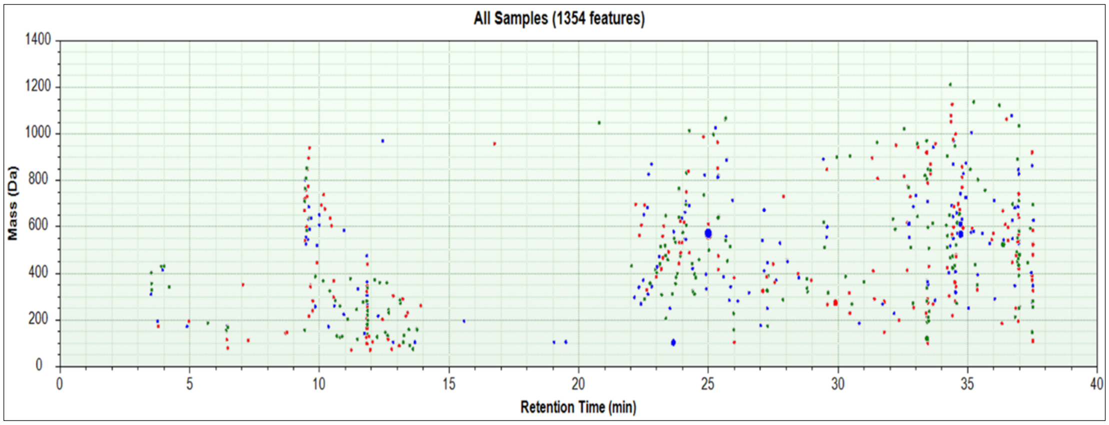
All Samples (1354 features)
Mass (Da)
Retention Time (min)

(a)

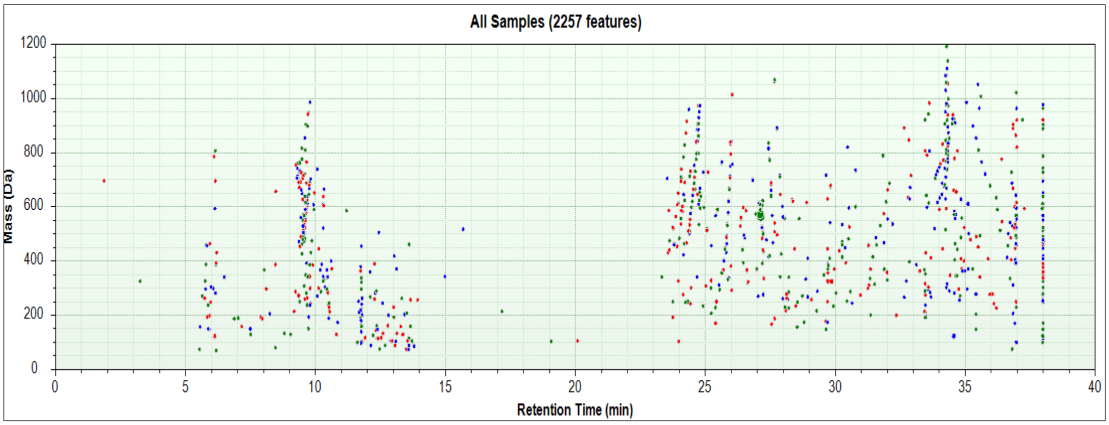
All Samples (2257 features)
Mass (Da)
Retention Time (min)

(b)

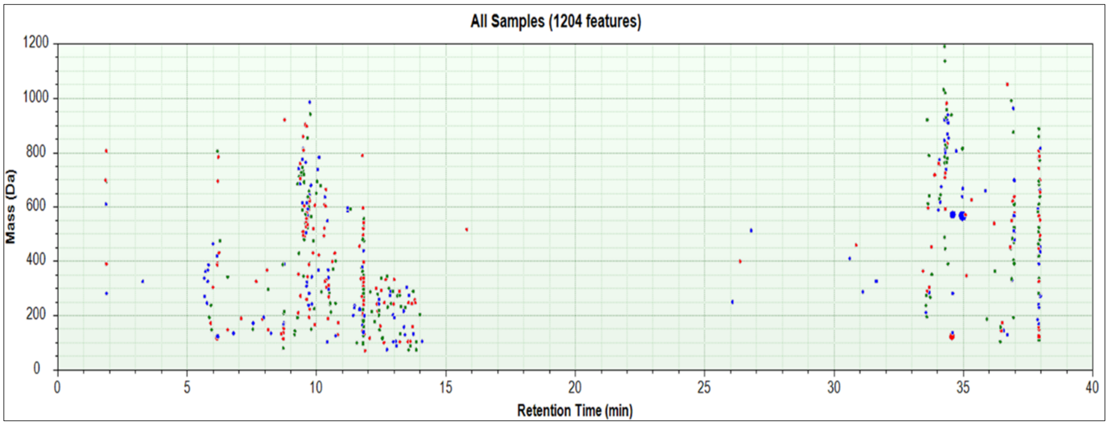
All Samples (1204 features)
Mass (Da)
Retention Time (min)

(c)

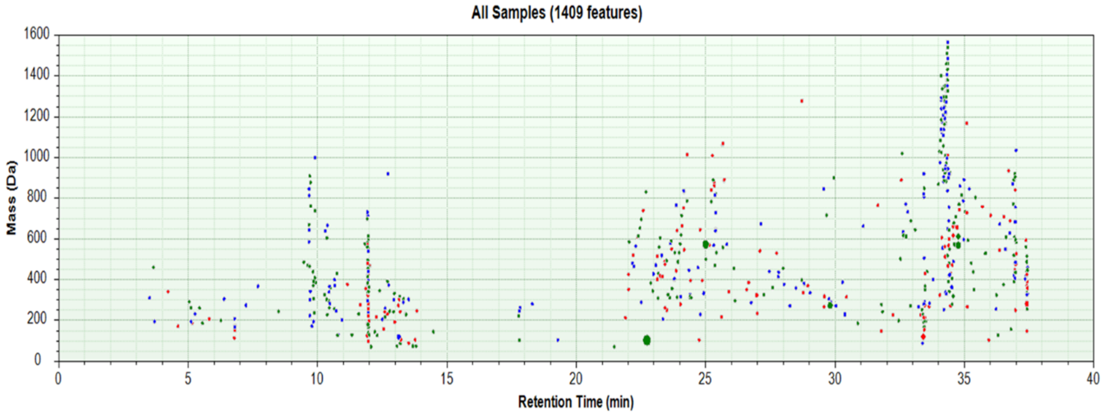

(d)

Figure S1. Retention time (RT)/Mass plot of the background was analyzed by RPLC-HILIC-ESI-TOF-MS in positive electrospray ionization mode. (a) 100% Methanol, (b)Acidic 90% methanol, (c) 50% Methanol, and (d) 100% Aqueous.

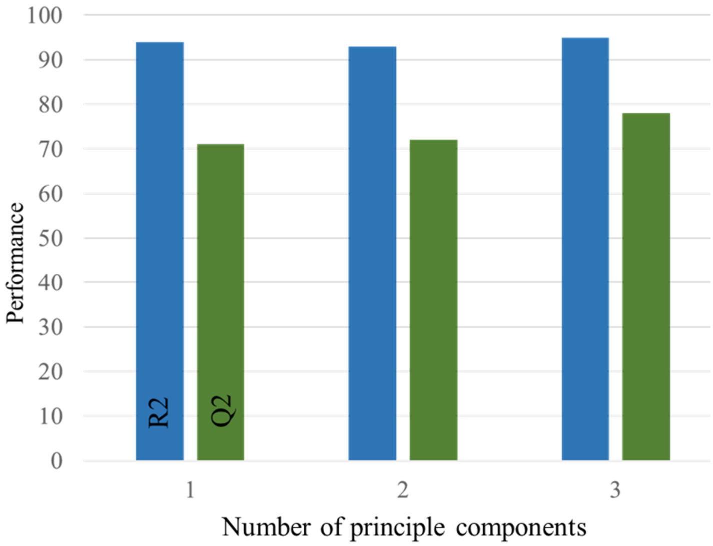

(a)

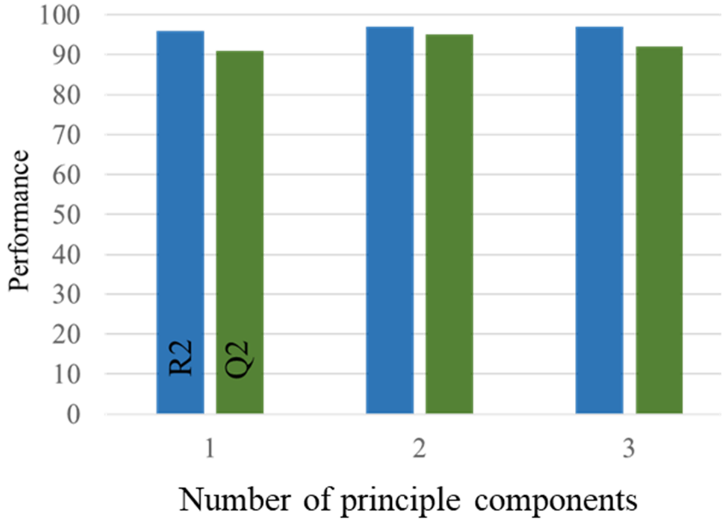

(b)

Figure S2. The Q^2/ R^2 Overview plot displays the individual cumulative R^2 (green columns) and Q^2 (blue columns) and Q^2 for the goodness of fits and cross-validation parameters (a) *P. australis* different parts. (b) *P. australis* different incubation

Diclofenac reference standards

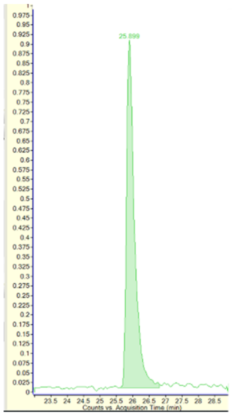

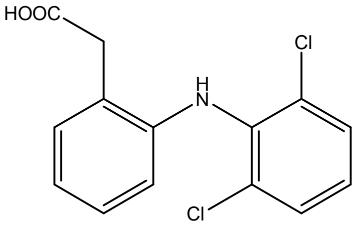

Diclofenac

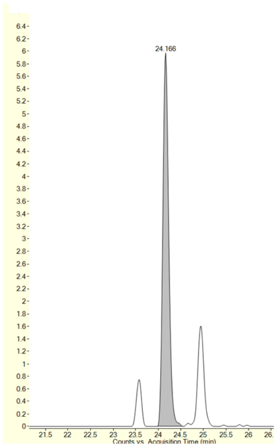

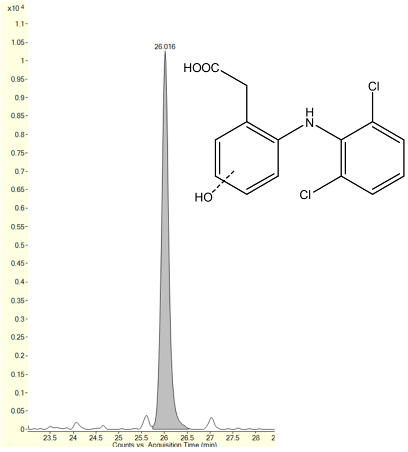

DM_1

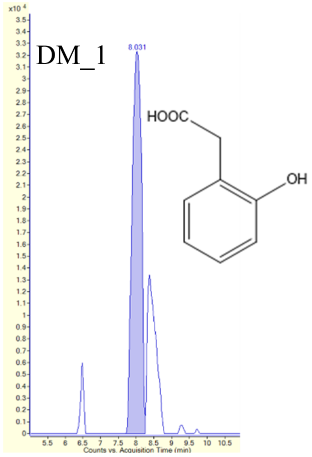

DM_2

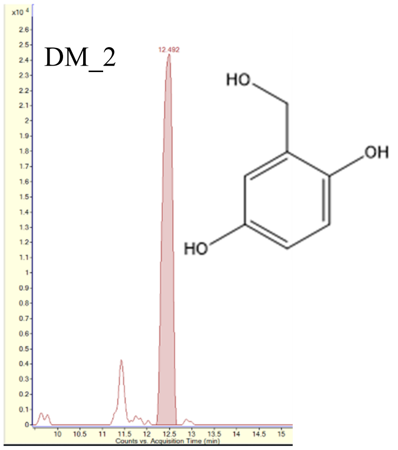

DM_3

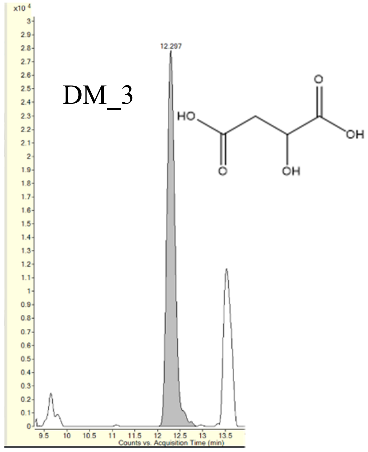

DM_4

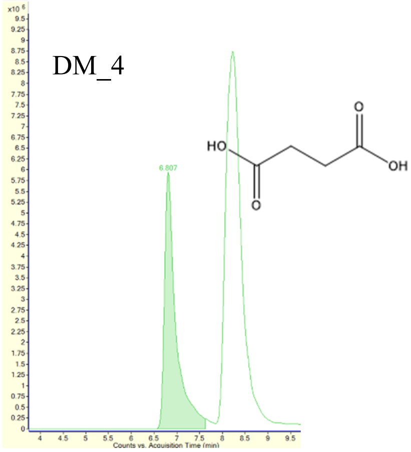

DM_5

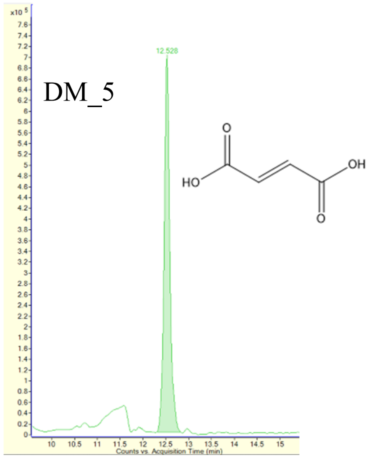

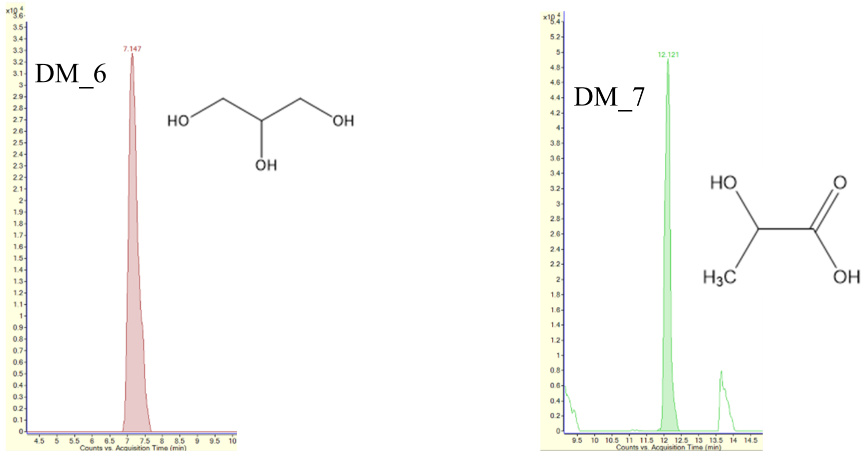

Figure S3. EICs were corresponding to measured diclofenac (right) and the reference standard (left), which were identified in the extracts of *Phragmites australis* leaf, rhizome, and roots incubated with 10 and 100 µM diclofenac. Also, EICs relative to transformed products are suspected in the extracts of *Phragmites australis* leaf, rhizome, and roots incubated with 10 and 100 µM diclofenac.

Supplementary information to the manuscript

	Standard reference EICs		*Phragmites* EICs
Carbamazepine-10,11-epoxide	Mass= 252.0903 Da		Mass= 252.0891 Da
	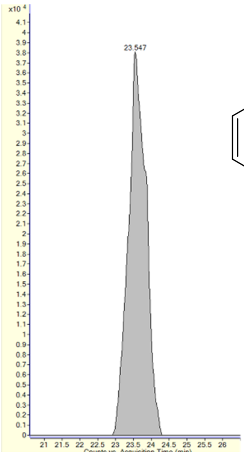	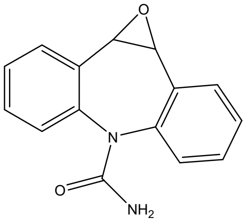	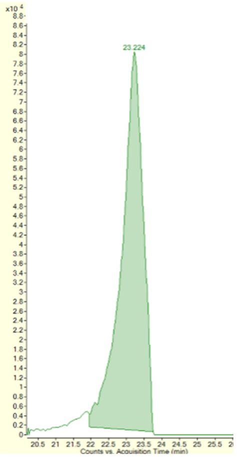
10,11-Dihydro-10,11-dihydroxy-carbamazepine	Mass=270.10 Da		Mass=270.0994 Da
	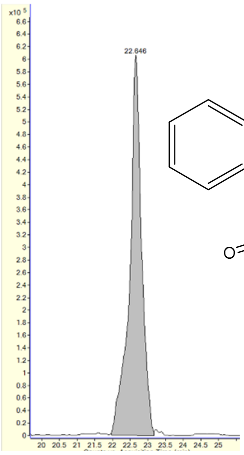	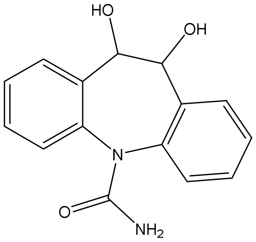	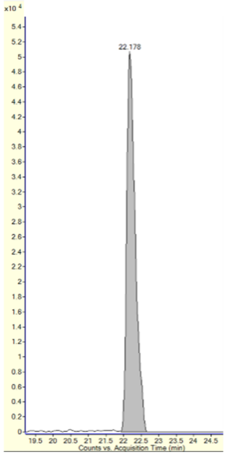
10,11-Dihydro-10-hydroxy-carbamazepine	Mass= 254.1055 Da		Mass= 254.1044 Da
	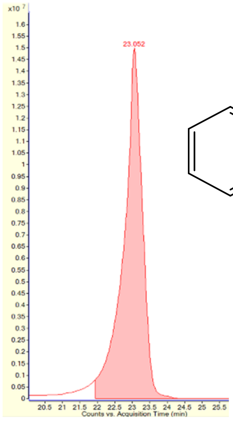	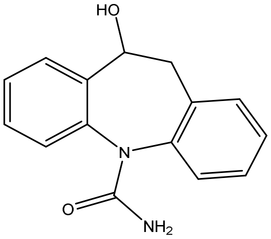	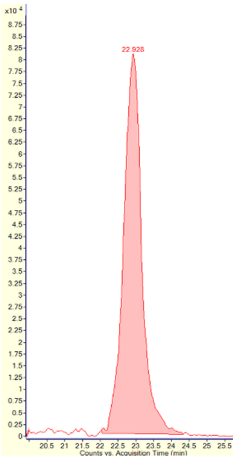

9-Acridine carboxaldehyde

Mass= 207.0684 Da

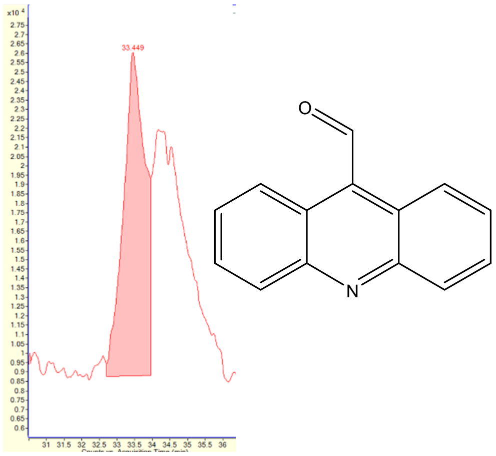

Mass=207.0682

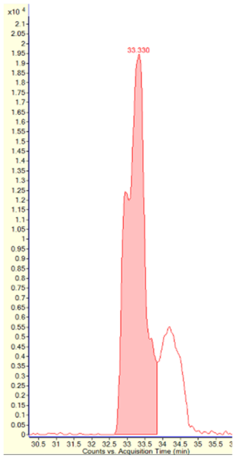

2,3-Dihydro-2,3-dihydroxy-carbamazepine

Mass= 270.1 Da

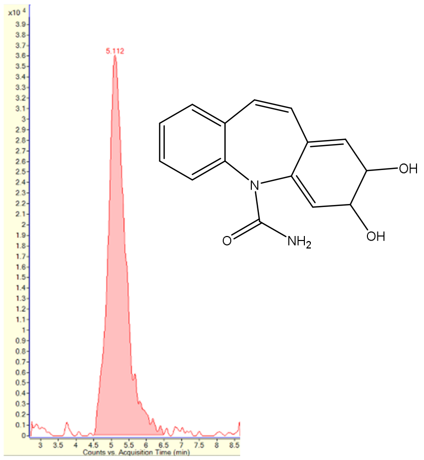

Mass= 270.0994 Da

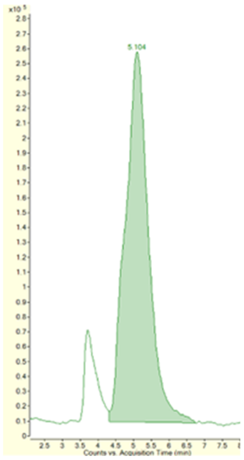

Figure S4. EICs were corresponding to carbamazepine (CBZ) and its transformed product standards (left), which were identified in the extracts of *Phragmites australis* leaf, rhizome, and roots incubated with 10 and 50µM carbamazepine (measured right).
